# Differential effects of multiplex and uniplex affiliative relationships on biomarkers of inflammation

**DOI:** 10.7717/peerj.19113

**Published:** 2025-03-24

**Authors:** Jessica J. Vandeleest, Lauren J. Wooddell, Amy C. Nathman, Brianne Beisner, Brenda McCowan

**Affiliations:** 1 California National Primate Research Center, University of California, Davis, Davis, CA, United States of America; 2 Department of Neurosurgery, Emory University, Atlanta, GA, United States of America; 3 Emory National Primate Research Center Field Station, Emory University, Lawrenceville, GA, United States of America

**Keywords:** Affiliation, Cytokines, Inflammation, Networks, Grooming, *Macaca mulatta*

## Abstract

Social relationships profoundly impact health in social species. Much of what we know regarding the impact of affiliative social relationships on health in nonhuman primates (NHPs) has focused on the structure of connections or the quality of relationships. These relationships are often quantified by comparing different types of affiliative behaviors (*e.g.*, contact sitting, grooming, proximity) or pooling affiliative behaviors into an overall measure of affiliation. However, it is unclear how the breadth of affiliative behaviors (*e.g.*, how many different types or which ones) a dyad engages in impact health and fitness outcomes. We used a novel social network approach to quantify the breadth of affiliative relationships based on two behaviors: grooming and sitting in contact. Dyadic relationships were filtered into separate networks depending on whether the pair engaged in multiple affiliative behaviors (multiplex networks) or just one (uniplex networks). Typically, in social network analysis, the edges in the network represent the presence of a single behavior (*e.g.*, grooming) regardless of the presence or absence of other behaviors (*e.g.*, contact sitting, proximity). Therefore, to validate this method, we first compared the overall structure of the standard network for each affiliative behavior: all grooming interactions regardless of contact sitting, and all contact sitting interactions regardless of grooming. We then similarly compared the structure of our filtered multiplex *vs.* uniplex networks. Results indicated that multiplex networks were more modular, reciprocal, and kin-based while connections in uniplex networks were more strongly associated with social status. These differences were not replicated when comparing networks based on a single behavior alone (*i.e.*, all grooming networks *vs.* all contact sitting networks). Next, we evaluated whether individual network position in multiplex *vs*. uniplex (novel approach) or grooming *vs*. contact sitting (traditional approach) networks differentially impact inflammatory biomarkers in a commonly studied non-human primate model system, the rhesus macaque (*Macaca mulatta*). Being well connected in multiplex networks (networks where individuals both contact sat and groomed) was associated with lower inflammation (IL-6, TNF-alpha). In contrast, being well connected in uniplex grooming networks (dyad engaged only in grooming and not in contact sitting) was associated with greater inflammation. Altogether, these results suggest that multiplex relationships may function as supportive relationships (*e.g.*, those between kin or strong bonds) that promote health. In contrast, the function of uniplex grooming relationships may be more transactional (*e.g.*, based on social tolerance or social status) and may incur physiological costs. This complexity is important to consider for understanding the mechanisms underlying the association of social relationships on human and animal health.

## Introduction

For decades, research has shown that social relationships impact individual health and fitness in a variety of animal species, with estimates of the magnitude of the association between social factors and mortality in humans on par with other well recognized mortality risks (*e.g.*, smoking, alcohol consumption) (Holt-Lunstad, Smith & Layton, 2010). While the importance of social factors for health and fitness are widely recognized, the mechanisms by which social life exerts its influence are still not well understood (McCowan et al., 2016; Ostner & Schülke, 2018). One reason is that social life is complex; it encompasses both agonistic and affiliative interactions that vary across a range of dimensions such as frequency of interaction, symmetry, tenor (friendly or hostile), predictability, and stability (Hinde, 1976a; Hinde, 1976b; Silk, Cheney & Seyfarth, 2013; Fischer et al., 2017). As such, new approaches addressing this complexity are needed to better understand the biological mechanisms by which social relationships influence health.

Two common methods for quantifying relationships and their association with health and fitness outcomes (*e.g.*, reproduction or lifespan) (Silk, Cheney & Seyfarth, 2013) include the use of sociality indices and social network analysis. The dyadic sociality index (DSI) is a commonly used measure of relationship quality in non-human primates. Relationships with high DSI scores (*e.g.*, strength of top three bonds or scores greater than the group mean) are thought to be of high quality and are commonly referred to as strong bonds and tend to be equitable, stable, involve frequent interaction, and are most common between kin and peers (Silk et al., 2010; Silk, 2014b; McFarland et al., 2017; Ostner & Schülke, 2018). In animals, a higher number or quality of these strong bonds has been associated with acute hormonal responses (*e.g.*, oxytocin or cortisol levels), increased reproduction, and longer survival (Crockford et al., 2013; Ostner & Schülke, 2018; Silk, Seyfarth & Cheney, 2018; Ellis et al., 2019). However, these strong affiliative bonds represent only a small fraction of the affiliative relationships an individual has (Silk et al., 2010; Schülke, Wenzel & Ostner, 2013; Fischer et al., 2017; McFarland et al., 2017; Silk, Seyfarth & Cheney, 2018) with weaker affiliative bonds comprising the remainder. The function of these other, weaker bonds has been hypothesized to increase social flexibility (*e.g.*, social connections can shift with environmental demands), allowing general social integration and indirect connections that might provide access to others who may have resources or information (McFarland et al., 2017; Silk, Seyfarth & Cheney, 2018). Yet, evidence for an association between weak bonds and health and fitness is mixed.

Social network analysis is a method well suited to quantifying the structure of social relationships and their potential to impact health and fitness. Typically, animal researchers use social network analysis to understand how one’s direct interactions with others or indirect connections (*e.g.*, friends of friends) impact a variety of health-related metrics and/or fitness outcomes. Direct connections measure the number of unique social partners one has (degree) or the frequency of social interactions (strength) which together have been proposed as measures of social integration (Ostner & Schülke, 2018; Ellis et al., 2019). For example, having more social partners has been associated with reduced likelihood of pathogen infection and fitness benefits (Balasubramaniam et al., 2016; McFarland et al., 2017; Brent, Ruiz-Lambides & Platt, 2017a). Indirect connections can be measured in a variety of ways that describe how one is connected in the broader social network ranging from measures of social embeddedness (closeness), cliquishness (clustering coefficient), and social bridging (betweenness). While the general pattern seen across these measures is that greater connectedness or centrality in specific behavioral networks is associated with lower risk for gastrointestinal pathogens, increased reproduction, and longevity (Brent et al., 2013; Balasubramaniam et al., 2016; Cheney, Silk & Seyfarth, 2016; Ostner & Schülke, 2018), there is little consistency across studies in identifying which specific network metric is important (Ostner & Schülke, 2018; Ellis et al., 2019; Snyder-Mackler et al., 2020). For example, eigenvector centrality, a measure of social capital (*i.e.,* one is well-connected to well-connected others) has been associated with health and fitness benefits in some studies (Brent et al., 2013; Balasubramaniam et al., 2016; Cheney, Silk & Seyfarth, 2016) but has been found to have no association in others (Brent et al., 2013; Balasubramaniam et al., 2016; Ellis et al., 2019).

While these approaches have proved fruitful, previous studies have either analyzed affiliative behaviors separately (*e.g.*, grooming *or* proximity) or lumped them together into a single index giving them roughly equal weight (*e.g.*, dyadic sociality index) (Silk, Cheney & Seyfarth, 2013; Ostner & Schülke, 2018). Unexplored is whether the diversity or breadth of affiliative behaviors exhibited in a relationship and the extent to which different types of affiliative behaviors are *integrated* (Hinde, 1976b, p. 197; Silk, Cheney & Seyfarth, 2013) which may provide key information as to the function or quality of a relationship. For example, a relationship in which a specific dyad engages in multiple types of affiliation (*e.g.*, contact sitting, grooming, *and* proximity) may differ from one in which a dyad engages in only one single behavioral domain (*e.g.*, contact sitting only, grooming only, *or* proximity only), even if the rate of interaction is the same. We may expect relationships identified as strong bonds (*e.g.*,  *via* DSI metrics) would likely also be those in which animals interact using multiple affiliative behaviors. It is somewhat unclear, however, what might be expected of relationships in which only one behavior is present, as the function of these relationships may be more closely tied to the specific behavior being measured (*e.g.*, thermoregulation might explain a relationship characterized by contact sitting only (Campbell et al., 2018), but this is unlikely to be a good explanation for proximity only). The choices animals make regarding how to interact with social partners (*i.e.,* the breadth of behaviors social partners engage in) may provide information about what function those relationships serve. In fact, Hinde (Hinde, 1976b) states that “the most important clues as to the significance and meaning of an interaction to the participants may be the context of other interactions in which it lies (p 4)”.

Recent advances in social network analysis and theory, and specifically multilayer or multiplex networks, (Beisner et al., 2020; *e.g.*, multiple types of interactions among the same set of individuals) may eventually provide a solution with tools to address such issues. However, currently, these analytical methods (*e.g.*, metrics like versatility and multiplex PageRank) are not sufficiently sophisticated to account for the many structural differences that exist between behavioral networks of different types (*e.g.*, proximity networks are often dense and undirected, contact networks may be more sparse and undirected, whereas grooming networks are directed) (Beisner et al., 2020). Yet, given the mounting evidence for the importance of multidimensionality in social relationships, the integration of diverse affiliative behaviors at this dyadic level may provide important additional information as to the quality or function of those relationships and their potential downstream impacts on health and fitness. Indeed, in human studies, multidimensional measures are often the strongest predictors of health and fitness (Berkman et al., 2000; Holt-Lunstad, Smith & Layton, 2010; Snyder-Mackler et al., 2020). Yet few animal studies to date (except see Fischer et al., 2017) have examined whether the integration of that diversity of interactions in a relationship provide clues as to the nature of those social relationships. One exception, conducted by Balasubramaniam et al. (2019), found that highly connected rhesus macaques in a grooming network were more likely to share E. coli species (a commensal gut bacteria), *but only if they were also well connected in a huddling network*, suggesting that the presence of multiple affiliative behaviors across an individual’s dyadic connections (*e.g.*, integration) may influence the potential transmission of gastrointestinal microbes.

Here we employed a social network approach to explicitly explore whether the integration of diverse affiliative behaviors (*i.e.,* grooming and contact sitting) within a relationship can provide new information regarding the quality and function of relationships and their impact on health-related outcomes in a commonly studied non-human primate model system, the rhesus macaque (*Macaca mulatta*). We use rhesus macaques as a group-living, nonhuman primate (NHP) model because their social relationships are highly differentiated, exhibit a high degree of complexity and individual variability, and have been linked to a variety of health and fitness outcomes (Vandeleest et al., 2016; Snyder-Mackler et al., 2016b; Brent, Ruiz-Lambides & Platt, 2017a; Capitanio, Cacioppo & Cole, 2019). Affiliation in primates takes many forms, including grooming, contact sitting, proximity, embracing, and coalitionary support (Sussman, Garber & Cheverud, 2005; Aureli & Schaffner, 2007; Pallante et al., 2019). In this study, we selected two of the most well studied affiliative behaviors in rhesus macaques, grooming and contact sitting, and explored whether relationships in which both were observed differed from those in which only a single behavior occurred. Grooming is the most widely studied affiliative behavior in rhesus monkeys and is used as a key indicator for the presence of affiliative relationships. Grooming has been proposed to serve multiple social functions including: to maintain social bonds (Dunbar, 1991) and social cohesion (Lehmann, Korstjens & Dunbar, 2007), and in exchange for tolerance from dominants, for agonistic support, or for access to resources (Noë & Hammerstein, 1995; Schino & Aureli, 2008; Borgeaud & Bshary, 2015; Snyder-Mackler et al., 2016a; Barrett & Henzi, 2006). Other affiliative behaviors in rhesus monkeys include spatial proximity, huddling, contact sitting, and alliances. Here we chose to use contact sitting behavior which is a combination of sitting in physical contact and huddling (contact sitting with trunk-to-trunk contact or while sleeping (Ueno & Nakamichi, 2018) as a second indicator of affiliative relationships (Campbell et al., 2018). While less frequently studied than grooming, contact sitting and huddling are behaviors frequently used to aid in thermoregulation in cold climates and preferentially used among animals with close relationships (Bernstein & Mason, 1963; Ueno & Nakamichi, 2018; Campbell et al., 2018).

To assess the integration of behaviors within a relationship we took a traditional single behavioral network (*e.g.*, grooming) and filtered it into “multiplex” and “uniplex” networks (Table 1). We use the term “multiplex” to refer to networks in which edges reflect the co-occurrence of multiple affiliative behaviors and the term “uniplex” to refer to networks in which edges reflect only one specific type of interaction (*e.g.*, grooming *or* contact sitting, not both) (Hinde, 1976b; Berkman et al., 2000). We compared network structure between three pairs of networks. First, we compared uniplex grooming (dyads only groom; edge-weights reflect grooming frequency) to multiplex grooming (dyads groom and contact sit; edge-weights reflect grooming frequency). Our rationale for constructing multiplex and uniplex networks in this manner was inspired by Balasubramaniam et al. (2019) which identified the combination of grooming and huddling behavior to be especially relevant for sharing of gastrointestinal microbes. Further, this distinction could also be hypothesized to relate to the different functions of grooming (social bonding/cohesion *vs.* exchange for tolerance and resources may potentially reflect strong *vs.* weak social bonds). Then to ensure that observed structural differences reflected biological differences and were not artifacts of our novel network filtering procedure, we also compared uniplex and multiplex contact sitting as well as networks generated from all the grooming *vs.* all contact sitting interactions. These relationships may promote health through multiple pathways, including: by buffering individuals from the negative consequences of stressors (Cohen & Wills, 1985; Balasubramaniam et al., 2019) or by impacting general physiological processes related to anabolic repair and maintenance (Cacioppo & Hawkley, 2003). Therefore, we further explored whether an individual’s position in these paired networks (*i.e.,* uniplex grooming *vs.* multiplex grooming, uniplex contact sitting *vs.* multiplex contact sitting, and all grooming *vs.* all contact sitting) was differentially associated with biomarkers of inflammation (*i.e.,* serum pro-inflammatory cytokines), which are common, well-established indicators of individual health status (Uchino et al., 2012; Capitanio, Cacioppo & Cole, 2019). Our prediction was that individuals exhibiting more central roles in multiplex networks would show lower levels of inflammatory cytokines than those exhibiting more central roles in uniplex networks.

**Table 1:** Network descriptions.

**Network**	**Description**
All grooming	Includes all grooming interactions, regardless of contact sitting behavior, with edge weights reflecting grooming frequency.
All contact sitting	Includes all contact sitting interactions, regardless of grooming behavior, with edge weights reflecting contact sitting frequency.
Uniplex grooming	Includes dyads observed grooming but never sitting in contact, with edge weights based on grooming frequency. This network contains a subset of dyads from the all grooming network.
Multiplex grooming^a^	Includes dyads that engaged in both grooming and contact sitting behaviors, with edge weights reflecting grooming frequency. This network contains a subset of dyads from the all grooming network.
Uniplex contact sitting	Includes dyads observed sitting in contact but never grooming, with edge weights based on contact frequency. This network contains a subset of dyads from the all contact sitting network.
Multiplex contact sitting^a^	Includes dyads that engaged in both grooming and contact sitting behaviors, with edge weights reflecting contact sitting frequency. This network contains a subset of dyads from the all contact sitting network.

**Notes.**

a Dyads included in the multiplex networks are the same however the edge-weights differ.

## Materials and Methods

### Study system

Rhesus macaques live in large multi-male, multi-female social groups organized by rank and kinship (Thierry, Singh & Kaumanns, 2004). For females, rank is inherited from their mothers and generally all members of a matriline hold adjacent ranks (Berman, 1980) (although see Fushing et al., 2011). In contrast, males generally immigrate into a new social group and may enter at the bottom of the hierarchy, queueing for rank, or attain rank through direct competition (Georgiev et al., 2016). Rhesus macaque females form the core of the social group with affiliation between both kin and non-kin playing a key role in maintaining group stability (Chapais, Girard & Primi, 1991; Thierry, Singh & Kaumanns, 2004). Although male social bonds have important fitness outcomes in macaques generally (Schulke et al., 2010), male rhesus macaques engage in social affiliation far less frequently (Drickamer, 1976) and tend to be more socially isolated than females (Brent, Ruiz-Lambides & Platt, 2017b). Therefore, we focused our study on females, which we predict will be more strongly impacted by social bonds than males.

### Subjects and housing

Subjects were 248 breeding age (3 years and older) female rhesus macaques (*Macaca mulatta*) that were born at the California National Primate Research Center in Davis, California. Subjects lived in one of four large multigenerational and matrilineal social groups (*i.e.,* contained groups of animals related through a common female ancestor and unrelated animals) containing 100–200 mixed-sex individuals (Table 2), each housed in a 0.2 hectare outdoor enclosure. Subjects were fed commercial monkey chow and foraging enrichment twice daily. Fruits or vegetables were provided weekly. Water was available ad libitum.

**Table 2:** Group demographics.

**Group**	**Group size (adults)**	**N (adult females)**	**# of matrilines** ^a^	**Mean matriline** ^a^ ** size (SD)**
Group A	131 (101)	74	13	5.7 (3.6)
Group B	204 (101)	67	33	2.0 (1.0)
Group C	125 (55)	39	6	6.5 (3.9)
Group D^b^	185 (96)	68	13	5.2 (2.3)

**Notes.**

a Number of matrilines and matriline size statistics include only breeding age females. Individuals were considered part of the same matriline if they could be traced back to the same female common ancestor at the time of group formation.

b Animals in these groups were designated as specific pathogen free (SPF).

### Behavioral data collection

Subjects were part of a larger study on the associations between social networks and health. Each group was studied for six continuous weeks. Groups A and B were studied during the birthing season from March to April 2013 and 2014, respectively. Groups C and D were studied during the breeding season from September to October 2013 and 2014, respectively. Behavioral data were collected six hours per day, four days per week from 0900–1200 and 1300–1600 each day by three observers (inter-rater reliability, Krippendorff’s alpha ≥ 0.85). Affiliative behavior was collected by one observer *via* scan sampling every 20-minutes (maximum 18 scans per day), where identities of all adult female dyads affiliating (*i.e.,* grooming or contact sitting) were recorded (Balasubramaniam et al., 2016). All animals had tattoos and fur markings that allowed accurate individual identification. All observers demonstrated animal ID reliability of >95%. Grooming was defined as cleaning or manipulating the fur of another animal and contact sitting included any body contact such as ventral contact, embrace, or side by side sitting for at least 3 s. During each scan, these behaviors were mutually exclusive for a dyad (an individual grooming another was not contact sitting with that individual). Affiliation scans produced 1,637 scans (Group A: *N* = 418, Group B: *N* = 410, Group C: *N* = 378, Group D: *N* = 431) and a median of 38 grooming interactions per female (group range 23–49) and 28 contact sitting interactions (group range 13–52). This sampling scheme has been shown to produce sufficiently sampled grooming and contact sitting networks (Balasubramaniam et al., 2018b). Because social status has been shown to impact inflammation (Snyder-Mackler et al., 2016b) (although see Vandeleest et al., 2016), dyadic aggression data was used to calculate dominance ranks and dominance certainty *via* the R package *Perc* (Fujii et al., 2016; Vandeleest et al., 2016). Aggression data (threats, chases, bites) were collected *via* an event sampling protocol for six hours per day, four days per week by two other observers (average of 42.5 interactions per individual, group range 36.2–51.9). Dominance rank was expressed as the percent of animals in the group outranked and therefore ranged from 0 (low) to 1 (high).

### Affiliative network analysis

First, weighted networks were constructed from all grooming and contact sitting interactions (Fig. S1A). Each of these networks (*i.e.,* all grooming or all contact sitting) were then separated into two more networks, a multiplex network where edges between dyads that both groomed and contact sat were retained (Figs. S1C or S1E), and a uniplex network in which edges were retained for dyads that only groomed (Fig. S1D) or only contact sat (Fig. S1B). Edge-weights in contact sitting networks (all contact sitting, uniplex contact sitting, multiplex contact sitting) reflected the rate at which a dyad was observed contact sitting over the 6-week period (*i.e.,* frequency of interaction/# scans where both animals were present; see Fig. S1 for sample edgelist for a tabular illustration of the network filtering procedure). Edge-weights in grooming networks reflected the number of unique scans a dyad was observed grooming (Table S1) divided by the number of scans in which both animals were known to be in the social group.

For each of the 6 networks (all grooming, multiplex grooming, uniplex grooming, all contact sitting, multiplex contact sitting, uniplex contact sitting) overall network structure was examined to determine if they exhibited differences in key structural features of rhesus relationships. Networks were treated as weighted and directed (grooming only) networks and metrics were calculated in R (ver. 4.0.5; R Core Team, 2021) using *igraph* (ver. 1.3.0). For example, evidence suggests that despotic macaques such as rhesus, particularly in large groups, are likely to have grooming networks that are modular (*i.e.,* shows subgrouping), expected to be based on kinship, and have individual network positions (*i.e.,* eigenvector centrality) that are correlated with rank (Sueur et al., 2011; Balasubramaniam et al., 2018a). Therefore, we examined whether these two networks differed in the degree of clustering (Newman’s modularity, clustering coefficient), kin bias (*e.g.*, proportion of kin (kin partners/total social partners)), and associations with rank (proportion of grooming up the hierarchy, rank disparity among grooming dyads) for each of the four groups studied. Also, because previous research has focused on bond strength, we further examined reciprocity, strength of relationships (average edge weight), and distribution of grooming (eigenvector centralization) across these network types.

Next, individual level centrality and cohesion measures were calculated in R (ver. 4.0.5; R Core Team, 2021) using *igraph* (ver. 1.3.0) for each of the six networks. The effects of the direct connections for individuals were measured using degree centrality and strength. The effect of an individual’s indirect connections in the network was evaluated using eigenvector, betweenness, and closeness centralities (Balasubramaniam et al., 2016; Balasubramaniam et al., 2019; Cheney, Silk & Seyfarth, 2016; Ostner & Schülke, 2018). In addition, the degree to which individuals were part of cohesive local communities was measured by the local clustering coefficient (*i.e.,* triadic closure). Multiple metrics were chosen to reflect the many different ways social integration can manifest (*e.g.*, bridging, cohesion, embeddedness, *etc*. Table 3). Additionally, we select a wide range of network measures due to the fact that key aspects of network structure (*e.g.*, community structure) can impact the relationship between network metrics (Farine & Whitehead, 2015; source article Box 3). Therefore, understanding which social network metrics may be particularly influential on health may require examination and comparison of multiple metrics to note whether certain metrics stand out as uniquely important (Wasserman & Faust, 1994; Makagon, McCowan & Mench, 2012; VanderWaal et al., 2014; Meghanathan, 2015; Farine & Whitehead, 2015; Grando, Noble & Lamb, 2016).

**Table 3:** Network metric definitions.

**Measure**	**Description**
Degree/Strength	Measures the *number* (unweighted) of partners or *frequency* of interaction (*i.e.*, strength) for each node (Sosa, Sueur & Puga-Gonzalez, 2020).
Eigenvector	Measures whether individuals are well connected to others that are also well connected, a measure of *social capital* (Sosa, Sueur & Puga-Gonzalez, 2020).
Betweenness	Measures the number of times a node lies on the shortest path between other nodes, which reflects an individual’s role in connecting others in the network or acting as a *social bridge* (Sosa, Sueur & Puga-Gonzalez, 2020).
Closeness	Measures how close each node is to all other nodes within the network, which reflects how *embedded* an individual is in the network (Kim, 2019).
Clustering Coefficient	Measures the extent to which a node’s neighbors are also connected to each other, a measure of *cliquishness or subgrouping* (Croft, James & Krause, 2008).

### Biological sample collection

Blood samples were taken during the fifth week of each group’s study period during routine, semi-annual health checks. On a single morning, all animals in a group were lightly sedated with ketamine (10 mg/kg) and given veterinary exams. Blood samples were obtained from the femoral vein and serum was aliquoted and stored at −80 °C for later assay. The order in which animals were processed and samples were collected was recorded to control for any potential impacts of the sampling procedure on the physiological variables examined.

### Pro-inflammatory cytokines

Chronic inflammation is associated with a variety of diseases (*e.g.*, diabetes, cardiovascular disease, cancer) and mortality (Bruunsgaard et al., 2003; Gan et al., 2004; Holmes et al., 2009), and high levels of pro-inflammatory cytokines, such as IL-6 and TNF-α, have previously been reported to be associated with social variables (*e.g.*, low social status, low social integration, poor quality relationships, loneliness) in both humans and rhesus macaques (Marucha et al., 2005; Friedman et al., 2005; Uchino et al., 2012; Vandeleest et al., 2016; Capitanio, Cacioppo & Cole, 2019). Therefore, we chose to measure serum levels of IL-6 and TNF-α as a general biomarker of health. Serum levels of IL-6 and TNF-α were measured simultaneously using commercially available, species specific Milliplex multi-analyte profiling (MAP) reagents purchased from EMD/Millipore (Billerica, MA, USA), and utilizing Luminex Xmap technology (Luminex, Austin, TX, USA). Color coded polystyrene microbeads coated with specific antibodies for IL-6 and TNF-α were incubated with the serum samples, washed, and then further reacted with biotinylated detector antibodies followed by Streptavidin-PE to label the immune complexes on the beads. After a final washing to remove all unbound material, the beads were interrogated in a BioPlex dual laser (BioRad, Hercules, CA, USA). The median fluorescent index for each sample was compared to a standard curve to calculate the concentration (IL-6: mean = 12.55 pg/mL, sd = 46.92, range = 0–690; TNF-α: mean = 185.0 pg/mL, sd = 442.27, range = 0–4052; see Fig. S2 for histograms). Samples were tested in duplicate and had an intra-assay coefficient of variability of 15.3%. Samples were re-analyzed if the CV was greater than 25% for all analytes measured. Manufacturer provided quality control samples fell within recommended ranges for all assays. Samples falling below the threshold sensitivity of the assay (1.6 pg/mL) were assigned a value of zero (IL-6: *N* = 77, TNF-α: *N* = 56).

### Statistical analysis

Two sets of analyses were done to determine whether (1) multiplex *vs* uniplex grooming networks, multiplex *vs* uniplex contact sitting networks, or all grooming and all contact sitting networks differ in structure and relationships to known social features of rhesus macaques (*e.g.*, kin bias, hierarchical organization), and (2) whether network metrics from these networks predicted biomarkers of inflammation, with a specific focus on the relative impact of multiplex *vs.* uniplex (or grooming *vs.* contact sitting) network position on inflammation.

First, due to the small sample size, paired t-tests were used to evaluate if network structure consistently differed across groups. Specifically, the structure of uniplex grooming *vs.* multiplex grooming, uniplex contact sitting *vs.* multiplex contact sitting, and all grooming *vs.* all contact sitting networks were compared for each social group. Network metrics examined included the degree of clustering (Newman’s modularity, clustering coefficient), kin bias (*e.g.*, proportion of kin (kin partners/total social partners)), associations with rank (proportion of grooming up the hierarchy, rank disparity among grooming dyads), reciprocity, strength of relationships (average edge weight), and distribution of grooming (eigenvector centralization) across these network types. Normality of the differences was evaluated using the Shapiro–Wilk test, and if significant then Wilcoxon signed rank tests were used. As a final structural analysis, we examined the correlations between individual level network positions from these two network types (Fig. S3) to evaluate multicollinearity within networks and associations between networks.

Next, to determine if an individual’s position in the multiplex or uniplex networks was associated with pro-inflammatory cytokines, we used an Information Theoretic (Burnham & Anderson, 2002) approach and ran generalized linear models using a negative binomial distribution (R package lme4 v.1.1-34) on each biomarker separately (see Vandeleest et al., 2016 for details on distribution choice and Fig. S2 for distributions). For these analyses, networks were treated as weighted but undirected because of our focus on the qualities of a relationship rather than focused specifically on grooming behavior and because contact sitting is recorded as an undirected behavior (*i.e.,* information on who initiated the interaction is unavailable). One animal was excluded from the IL-6 analyses because it was an outlier with influence (Cook’s D > 1); all other outliers had a Cook’s D < 0.5 and therefore were included in the analyses. Animals that were unconnected in any networks were assigned a score of 0 for all network metrics (1 animal in uniplex grooming, 6 animals in uniplex contact sitting. Model building proceeded in two steps for each outcome (*i.e.,* IL-6, TNF-α). For all steps, ΔAIC > 2 was used to identify potential predictors and candidate models. First, variables from the literature (age, dominance rank, dominance certainty, sampling order (Capitanio, Mendoza & McChesney, 1996; Bruunsgaard, Pedersen & Pedersen, 2001; Vandeleest et al., 2016; Rohleder, 2019)), although not of direct interest here, were evaluated to determine if it was necessary to control for their effects on inflammation before examining social network variables. Next, due to the exploratory nature of our analysis and our goal of directly comparing effects of centrality in different networks and predictions for multiplex *vs* uniplex networks, we developed a comprehensive set of models and evaluated AIC to identify a set of candidate models. To balance between exploration of the model space and model dredging (Fraser et al., 2018) we only ran models comparing metrics from (1) uniplex and multiplex grooming, (2) uniplex and multiplex contact sitting networks, (3) grooming *vs* contact sitting networks. This resulted in 110 models being run for each outcome measure (see Tables S3, S5), and a set of candidate models was identified (ΔAIC ≤ 2). We restrict our analysis to this subset of possible models because our primary goal was to compare uniplex and multiplex networks derived from the grooming network since grooming is the most commonly studied affiliative behavior. However, due to the novelty of this method and to allow for more direct comparison to the more common approaches of networks derived from single behaviors we also included models that compared multiplex *vs* uniplex contact sitting centrality and grooming *vs* contact sitting centrality metrics. Additionally, due to the large number of models run we also evaluated effects in a set of models with a more liberal cutoff (ΔAIC ≤ 7) to evaluate the robustness and consistency of effects across this wider set of models (Tables S4, S6).

### Ethical note

All procedures used in this study met all legal requirements of the United States as well as guidelines set by the American Society of Primatologists regarding the ethical treatment of non-human primates. This study was approved by the Institutional Care and Use Committee at the University of California, Davis and was carried out in compliance with the ARRIVE guidelines.

## Results

### Multiplex *vs.* uniplex affiliation networks

For all groups studied, clear differences in network topology, kinship, and associations with dominance rank were seen between the multiplex and uniplex affiliative networks (Table 4). Multiplex grooming networks had higher average edge-weight (the average interaction rate per social partner), clustering coefficient, and modularity (the degree to which the network can be divided into subgroups) than uniplex grooming networks for all groups. Notably, although average edge-weights in the multiplex networks were higher than uniplex networks, the predominant number of interactions observed per dyad for all networks was 1–2 interactions (Fig. S4). Multiplex grooming networks also consistently showed more kin bias (proportion kin) and reciprocity than uniplex grooming networks. Uniplex grooming networks were more strongly organized around social status with grooming more likely to be directed up the hierarchy and a greater rank disparity between grooming partners than in the multiplex grooming networks. Results for the multiplex *vs* uniplex contact sitting networks were the same as for multiplex and uniplex grooming networks (Table 4, Table S2). In contrast, the structure of the all grooming and all contact sitting networks did not differ, with the exception of average edge-weight which was higher in contact sitting networks and modularity which was higher in the grooming networks.

**Table 4:** Whole network metric comparisons.

**Network**	**Multi *vs.* uni grooming**	**Multi *vs.* uni contact sit**	**Contact sit *vs* grooming**
Density	Multi = Uni	Multi = Uni	CS = GR
Modularity	Multi > Uni^*^	Multi > Uni^*^	CS < GR^*^
Eigenvector centralization	Multi = Uni	Multi = Uni	CS = GR
Avg edge weight	Multi > Uni	Multi > Uni	CS > GR^*^
Clustering coefficient	Multi > Uni^*^	Multi > Uni^*^	CS = GR
Reciprocity	Multi > Uni^*^	–	–
Proportion kin	Multi > Uni^*^	Multi > Uni^*^	CS = GR
Proportion up rank	Multi < Uni^*^	–	–
Rank disparity	Multi < Uni^*^	Multi < Uni^*^	CS = GR
Rank/eigenvector centrality correlation	Multi = Uni	Multi = Uni	CS = GR

**Notes.**

Multi Multiplex network Uni Uniplex network GR Grooming CS Contact Sit

Effect indicates the overall difference between networks for all groups using a paired *t*-test. See Table S2 for exact statistics.

* *p* < 0.05.

### Relationship dimensionality and biomarkers of inflammation

**Figure 1:**
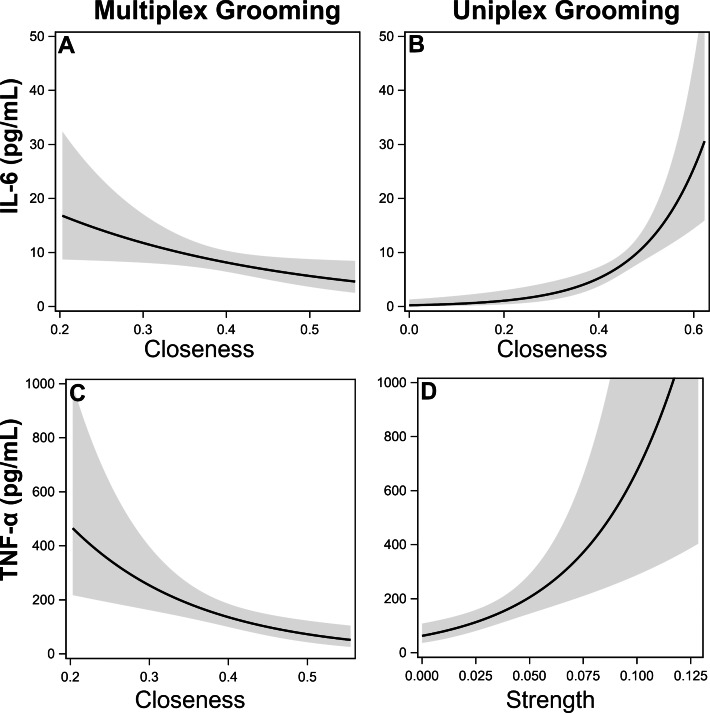
Effects of multiplex and uniplex grooming centrality on inflammatory cytokines. Effects of multiplex (Multi) grooming closeness (A) and uniplex (Uni) grooming closeness (B) on levels of IL-6 with 95% confidence intervals (Model 1). Effects of multiplex grooming closeness (C) and uniplex grooming strength (D) on levels of TNFα with 95% confidence intervals (Model 1).

**IL-6.** There was a single top model identified which included uniplex and multiplex grooming closeness (Table 5). Specifically, individuals that were more central (*i.e.,* higher closeness) in the uniplex grooming network exhibited higher inflammation while the opposite was true for multiplex grooming (Figs. 1A–1B).

**Table 5:** Candidate model outputs.

	**IL-6**	**TNF-α**	
**Model parameters**	**Model 1**	**Model 1**	**Model 2**
Intercept	−0.102	6.53^**^	4.83^**^
Uni GR closeness	7.92^**^	–	–
Uni GR strength	–	23.81^**^	25.36^**^
Multi GR closeness	−3.68^*^	−6.26^**^	–
Multi GR degree	–	–	−0.12^**^
ΔAIC	0	0	1.59
Model Weight	1	0.689	0.311

**Notes.**

* *p* < 0.05.

** *p* < 0.01.

Uni Uniplex Multi Multiplex GR Grooming network

All models were run using a negative binomial distribution and included a random effect of group.

**TNF-α.** There were two candidate models identified; both of which included comparisons of multiplex and uniplex grooming centrality (Table 5). As with IL-6, lower centrality in the multiplex grooming network (degree or closeness) but higher centrality in the uniplex grooming network (strength) were consistently associated with higher levels of TNF-α (Figs. 1C–1D).

## Discussion

Social primates have a complex web of differentiated social relationships, which vary in their quality, structure, and function. While strong affiliative social relationships are usually associated with better health, less is known on how the multidimensionality or integration of different affiliative behaviors within a social relationship might impact health. We identified affiliative relationships that were multiplex (dyads affiliated using both grooming and contact sitting behavior) *versus* uniplex (dyads only groomed or only contact sat). Examination of these networks revealed that they differed in topology, kinship, and associations with rank. Multiplex networks were more modular, clustered, reciprocal, had higher average edge weights, and were more strongly associated with kinship. In contrast, dyads in the uniplex networks tended to be of more disparate ranks. Notably, these differences in kinship and rank between multiplex and uniplex networks were not apparent when looking at all grooming or contact sitting interactions. The health impacts of these two networks differed as well, with females that were *less* socially connected in multiplex grooming networks exhibiting higher levels of inflammatory cytokines (IL-6 and TNF-α), whereas females *more* socially connected in uniplex grooming networks exhibited higher levels of inflammatory cytokines. Notably, these effects were primarily seen in multiplex and uniplex grooming, not in the other networks tested. These results suggest that grooming that occurs in the context of multiplex affiliative relationships may result in health benefits (*i.e.,* reduced inflammation). In contrast, grooming occurring in uniplex affiliative relationships may result in health costs (*i.e.,* higher inflammation).

Networks consisting of dyads with multiplex relationships showed differences from uniplex relationships in network topology, kinship, and associations with dominance. Multiplex networks had structural characteristics consistent with strong bonds or supportive affiliative relationships (Silk et al., 2009; Silk et al., 2010; Silk, 2014a). Specifically, interactions in the multiplex networks were more likely to be reciprocal, frequent (*i.e.,* higher edge-weight), clustered, and associated with kinship, suggesting they are relationships that are regularly maintained and potentially more stable across time (Silk et al., 2010; Silk, 2014a). Previous methods demonstrating that strong bonds enhance fitness, particularly those using sociality indices, have also used multiple behaviors to assess relationship strength (*e.g.*, grooming and proximity (Fischer et al., 2017; McFarland et al., 2017; Silk, Seyfarth & Cheney, 2018)). However, these methods rely on total duration or frequency of affiliation to describe relationships rather than characterizing the breadth or dimensionality of the relationships (*e.g.*, dyads can have high DSI through grooming, proximity, or both). Similar to strong bonds, multiplex affiliative relationships may improve health and fitness by buffering individuals from the negative impacts of stress, improving predator detection, promoting offspring survival, and improving social stability (Beisner et al., 2011; Micheletta et al., 2012; Balasubramaniam et al., 2016; Cheney, Silk & Seyfarth, 2016).

Also consistent with the literature on strong affiliative bonds, being well connected to others was associated with biomarkers of better health. Specifically, the negative association between multiplex grooming centrality (*e.g.*, degree, closeness centrality) and biomarkers of inflammation indicated that individuals that were generally well connected in the network (*e.g.*, at the core of the group) may be at lower risk for inflammation related diseases (Bruunsgaard et al., 2003). Specifically, having more strong social partners (*i.e.,* degree) or being more embedded in a strongly bonded network (*i.e.,* closeness) could be particularly important. This possibility, however, requires replication due to the influence of network structure (*e.g.*, modularity) on the intercorrelations among individual level centrality metrics. Our results add to the literature suggesting that strong bonds may exert their effects by altering endocrine and immune function (Boccia et al., 1997; Shutt et al., 2007; Crockford et al., 2013; Snyder-Mackler et al., 2020) which could in turn lead to effects on fitness. Consistent with this idea, Yang et al., (2016) found in humans that socially integrated individuals (*i.e.,* those with more social connections across multiple domains) exhibited lower inflammation, whereas social strain (*e.g.*, higher levels of family criticism or demands) was associated with greater inflammation. While our study only examines a single time-point, given that familial and friend relationships tend to endure through extended periods, often persisting over decades (in both humans and NHPs), these relationships may have an important and long-lasting impact on health.

Uniplex grooming relationships may reflect relationships that are more transactional in nature (Silk, 2002). The fact that uniplex grooming relationships are less kin biased but likely to occur between dyads of more disparate ranks suggests that these relationships may be more related to grooming being used as a commodity in exchange for tolerance or support from higher ranking animals. These transactional relationships may reflect a desire to maintain peace/tolerance or used in a biological market exchange (Noë & Hammerstein, 1995; Schino & Aureli, 2008; Borgeaud & Bshary, 2015; Barrett & Henzi, 2006), rather than reflecting a strong affiliative relationship. The positive association between females’ connectedness in uniplex grooming networks and biomarkers of inflammation suggests that uniplex grooming relationships may not be supportive on their own and instead are associated with increased physiological costs, at least in the short term. Specifically, predictors of inflammation in the uniplex grooming networks included strength or closeness centrality. However, the various network centrality metrics from the uniplex grooming network were more highly correlated with each other than the other networks, and therefore it is difficult to identify which specific aspect of uniplex grooming centrality might be driving these effects. However, collectively this group of candidate predictors indicates that greater general connectedness (direct and indirect) in uniplex grooming was associated with increased inflammation. Uniplex grooming relationships were maintained through generally less frequent interactions that were more likely to occur between animals of disparate ranks which may result in greater uncertainty regarding the outcome of any given interaction. This uncertainty may be stressful, and therefore have at least short-term physiological costs (Schino & Alessandrini, 2015; Christensen et al., 2024). If these relationships are more transactional in nature, then maintaining more of these transactional relationships may result in increased stress, which if sustained can result in long-term physiological costs (Snyder-Mackler et al., 2020). It is possible that these short-term costs are actually investments that may manifest in future benefits (*e.g.*, tolerance, alliance support) that would offset these costs, yet this is difficult to test as the “commodities” exchanged may be heterogeneous and the time-scale for market exchanges is often unclear (Dunayer & Berman, 2016). However, other work points to benefits of weak or economically based bonds to survival and reproduction (McFarland et al., 2017; Ellis et al., 2019) (although see Silk, Seyfarth & Cheney, 2018). While these types of connections may have ultimate fitness benefits (*e.g.*, alliance support, increased access to food), this research suggests they may also be associated with proximate costs.

## Conclusion

Both humans and many species of NHPs engage in a complex interconnected system of social interactions. Understanding the mechanisms by which social relationships impact health and fitness remains a challenge. Decades of research has established that affiliative social relationships can benefit health, however, the complexity and multidimensionality of relationships has yet to be explored. By utilizing a network approach, we were able to characterize two types of affiliative social relationships that differed in their network topology, kin bias, associations with rank, and importantly their associations with biomarkers of inflammation. Our research has indicated that features of multiplex affiliative relationships are consistent with the concept of a strong supportive relationships and may support health and fitness. In contrast, more transactional affiliative relationships (*e.g.*, uniplex affiliation) may incur short-term health costs yet may result in ultimate benefits through commodity exchange. Still unclear is whether these effects are specific to the combination of behaviors used here (*i.e.,* contact sitting and grooming), or if other affiliative behaviors (*e.g.*, proximity) might provide similar information. Further research into the dimensionality of relationships might reflect different qualities or functions of relationships is needed. However, this complexity is important to consider for understanding the mechanisms underlying the impact of social relationships on human and NHP health.

## Supplemental Information

10.7717/peerj.19113/supp-1 Supplemental Information 1 Supplementary Tables and Figures

## Additional Information and Declarations

### Data Availability

The following information was supplied regarding data availability: The data are available at Dryad: Vandeleest, Jessica; Wooddell, Lauren; Nathman, Amy et al. (2024). Differential effects of multiplex and uniplex affiliative relationships on biomarkers of inflammation [Dataset]. Dryad. https://doi.org/10.5061/dryad.866t1g1xq.
