# Supplementary material for: Differential effects of multiplex and uniplex affiliative relationships on biomarkers of inflammation
Source: PeerJ. 2025 Mar 24;13:e19113. doi: 10.7717/peerj.19113 (PMC11949109; doi:10.7717/peerj.19113)
Supplement: Supplemental Information 1 [file peerj-13-19113-s001.pdf]

Differential effects of multiplex and uniplex affiliative relationships on biomarkers of inflammation

Supplementary Materials

Jessica Vandeleest[1*], Lauren J. Wooddell[2], Amy C. Nathman[1], Brianne A. Beisner[3], Brenda McCowan[1]

[1]California National Primate Research Center, University of California, Davis, CA, United States

[2]Department of Neurosurgery, Emory university, Atlanta, GA

[3]Emory National Primate Research Center Field Station, Lawrenceville, GA, United States

[*]Corresponding author details: Jessica Vandeleest, PhD

Email: vandelee@ucdavis.edu

Contents

Figure S1: Network filtering example

Figure S2: Cytokine histograms.

Figure S3. Network Correlation Heatmaps

Figure S4: Histograms of edge weights by network and social group.

Table S1: Network Node and Edge Details

Table S2: Paired comparisons of whole network metrics

Table S3: Model Building Log for IL-6

Table S4: Model results for top models: IL-6

Table S5: Model Building Log for TNF-α

Table S6: Model results for top models: TNF-α

Figure S1: Network Filtering example

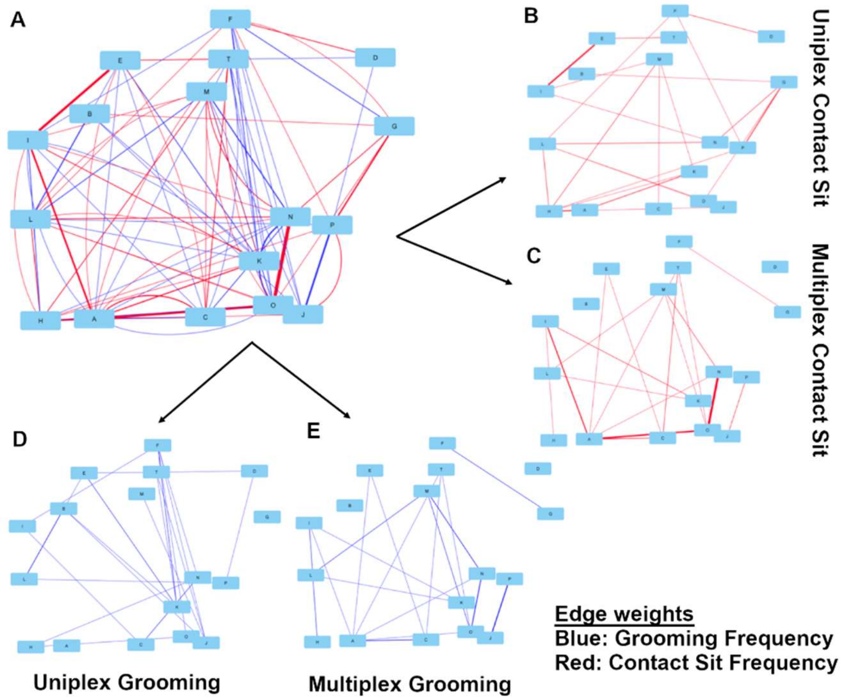

A network example using a subset of animals from Group C. Edge weight is represented using line thickness and behavior is indicated by color. Network filtering started with networks consisting of (A) all observed grooming (blue) and contact sitting (red) interactions. Edges from these networks were then filtered into multiplex grooming (E) or multiplex contact sitting (C) networks if dyads engaged in both behaviors. Dyads that only groomed were filtered into a uniplex grooming network (D) and dyads only engaged in contact sitting were filtered into a uniplex contact sitting network (B).

Traditional Single Behavior Networks				Networks defined by the presence/absence of multiple behaviors			
**All GR**	**All CS**	**ID1**	**ID2**	**Uni GR**	**Multi GR**	**Multi CS**	**Uni CS**
5	1	A	I	0	5	1	0
3	3	A	C	0	3	3	0
1	2	F	G	0	1	2	0
1	1	A	E	0	1	1	0
1	1	C	E	0	1	1	0
7	0	E	I	7	0	0	0
5	0	A	H	5	0	0	0
2	0	D	F	2	0	0	0
1	0	B	G	1	0	0	0
0	3	B	L	0	0	0	3
0	2	F	K	0	0	0	2
0	1	D	E	0	0	0	1
0	1	C	I	0	0	0	1

Colums on the left (All GR, All CS) represent the count of scans in which that behavior was observed for that specific dyad (ID1, ID2). These data are then filtered into Multiplex networks; dyads that were observed engaging in both behaviors were included in the Multiplex networks. While multiplex grooming and multiplex contact stitting networks share the same edges, their edge-weights for their networks are based on the count of behavior for their respective entworks (e.g., weights for Multi GR are based on the count of grooming, weights for Multi CS are based on the count of contact sitting). Uniplex networks only contain edges for dyads that engaged in that behavior and NOT the other. For example, dyads in the uniplex grooming network were observed grooming but never contact sitting.

Figure S2. Histogram of a) IL-6 and b) TNF-α. Plot a does not include the outlier that was excluded from data analysis.

a)

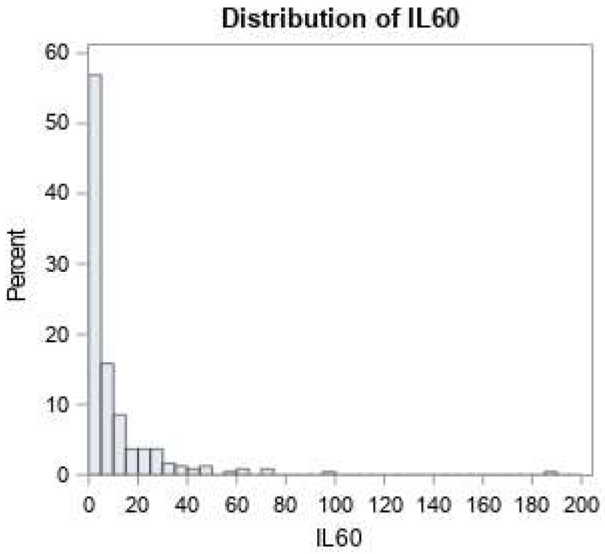

b)

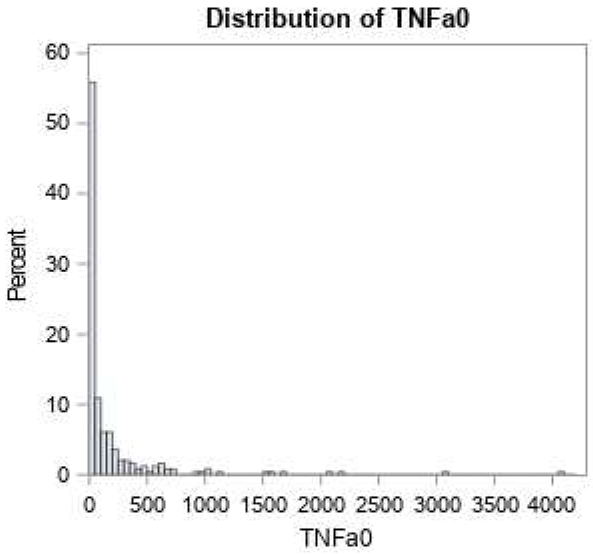

Figure S3: Network Correlation Heatmaps

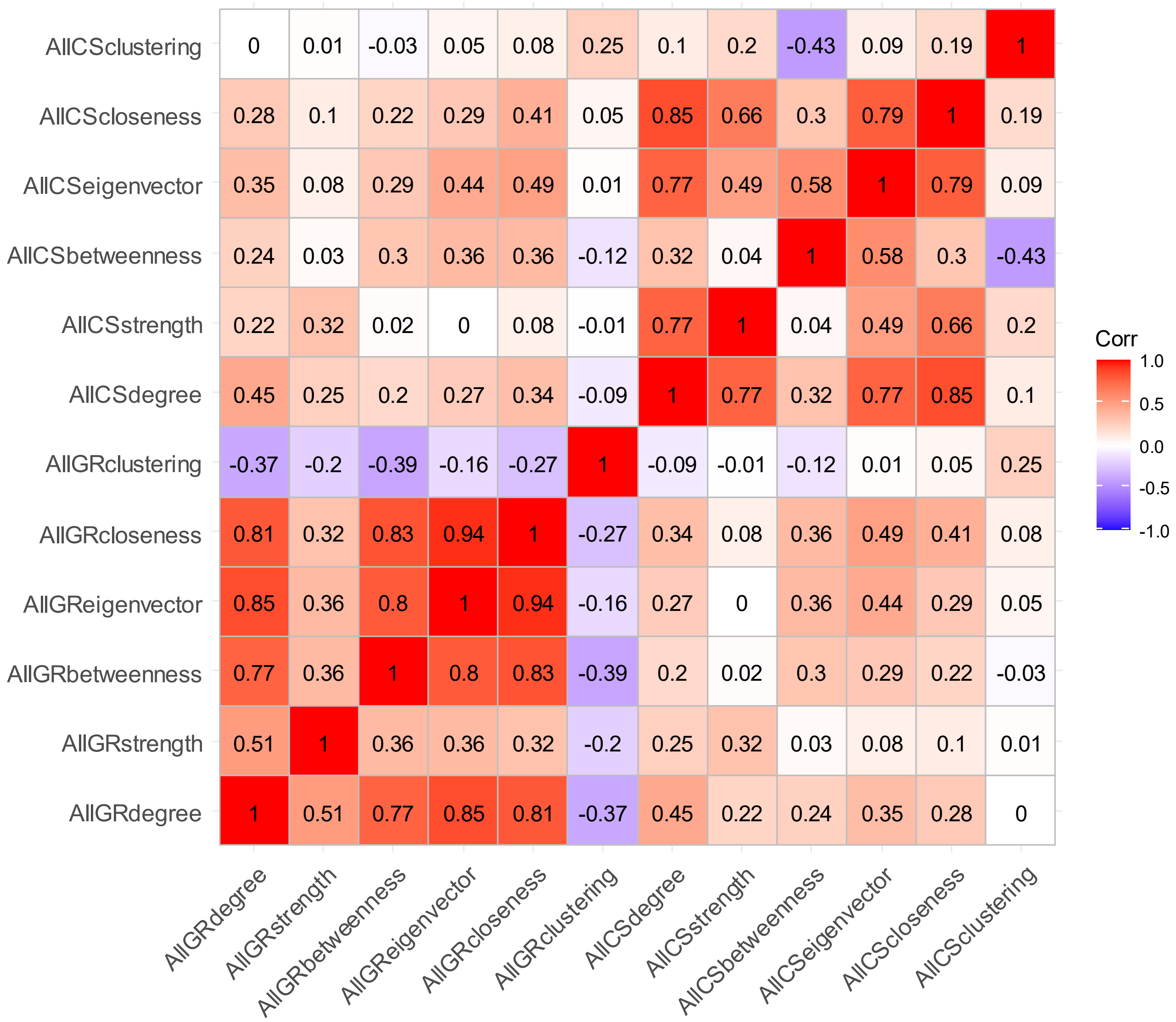

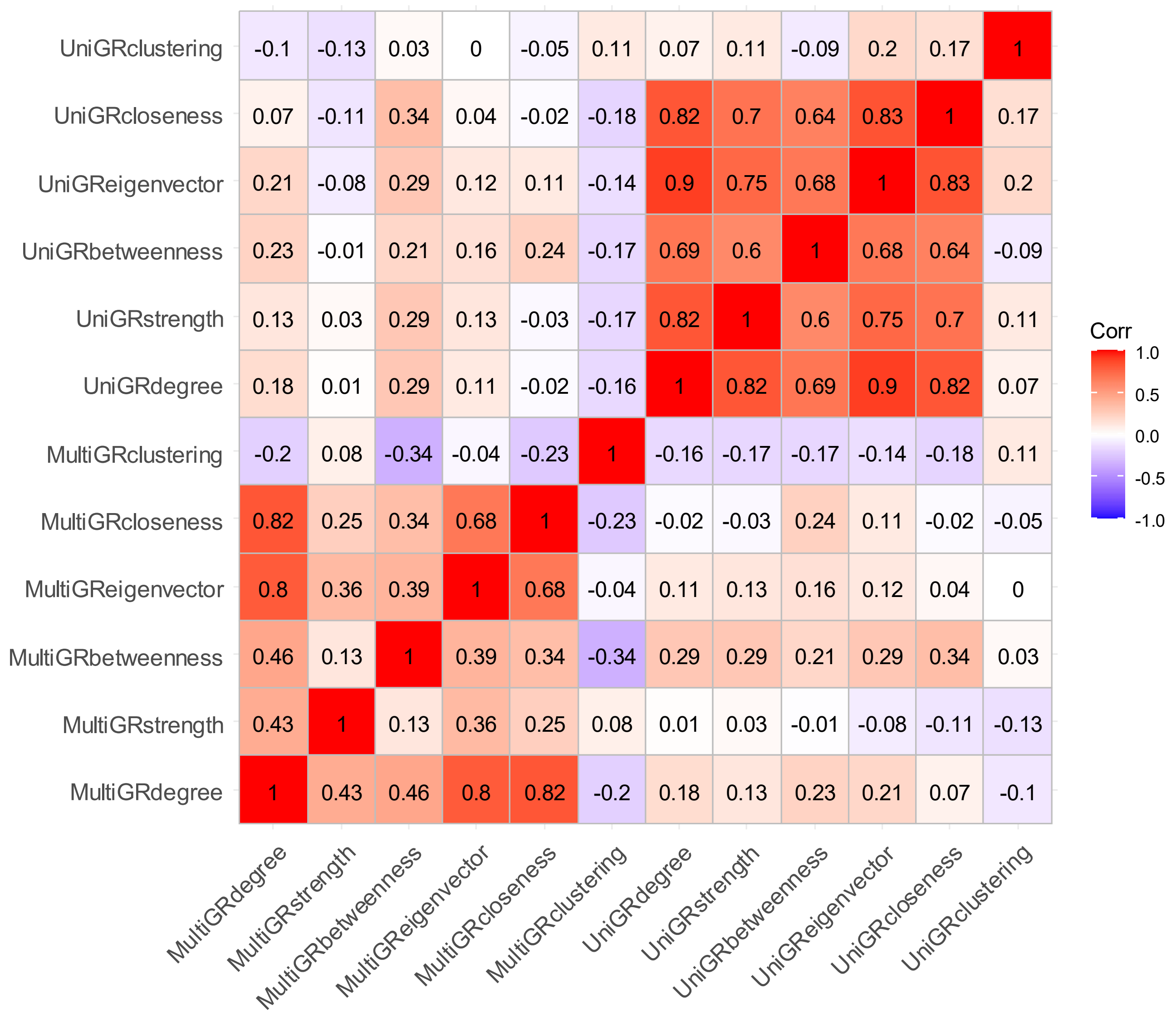
UniGRclustering
UniGRcloseness
UniGReigenvector
UniGRbetweenness
UniGRstrength
UniGRdegree
MultiGRclustering
MultiGRcloseness
MultiGReigenvector
MultiGRbetweenness
MultiGRstrength
MultiGRdegree
-0.1 -0.13 0.03 0 -0.05 0.11 0.07 0.11 -0.09 0.2 0.17 1
0.07 -0.11 0.34 0.04 -0.02 -0.18 0.82 0.7 0.64 0.83 1 0.17
0.21 -0.08 0.29 0.12 0.11 -0.14 0.9 0.75 0.68 1 0.83 0.2
0.23 -0.01 0.21 0.16 0.24 -0.17 0.69 0.6 1 0.68 0.64 -0.09
0.13 0.03 0.29 0.13 -0.03 -0.17 0.82 1 0.6 0.75 0.7 0.11
0.18 0.01 0.29 0.11 -0.02 -0.16 1 0.82 0.69 0.9 0.82 0.07
-0.2 0.08 -0.34 -0.04 -0.23 1 -0.16 -0.17 -0.17 -0.14 -0.18 0.11
0.82 0.25 0.34 0.68 1 -0.23 -0.02 -0.03 0.24 0.11 -0.02 -0.05
0.8 0.36 0.39 1 0.68 -0.04 0.11 0.13 0.16 0.12 0.04 0
0.46 0.13 1 0.39 0.34 -0.34 0.29 0.29 0.21 0.29 0.34 0.03
0.43 1 0.13 0.36 0.25 0.08 0.01 0.03 -0.01 -0.08 -0.11 -0.13
1 0.43 0.46 0.8 0.82 -0.2 0.18 0.13 0.23 0.21 0.07 -0.1
MultiGRdegree
MultiGRstrength
MultiGRbetweenness
MultiGReigenvector
MultiGRcloseness
MultiGRclustering
UniGRdegree
UniGRstrength
UniGRbetweenness
UniGReigenvector
UniGRcloseness
UniGRclustering
Corr
1.0
0.5
0.0
-0.5
-1.0

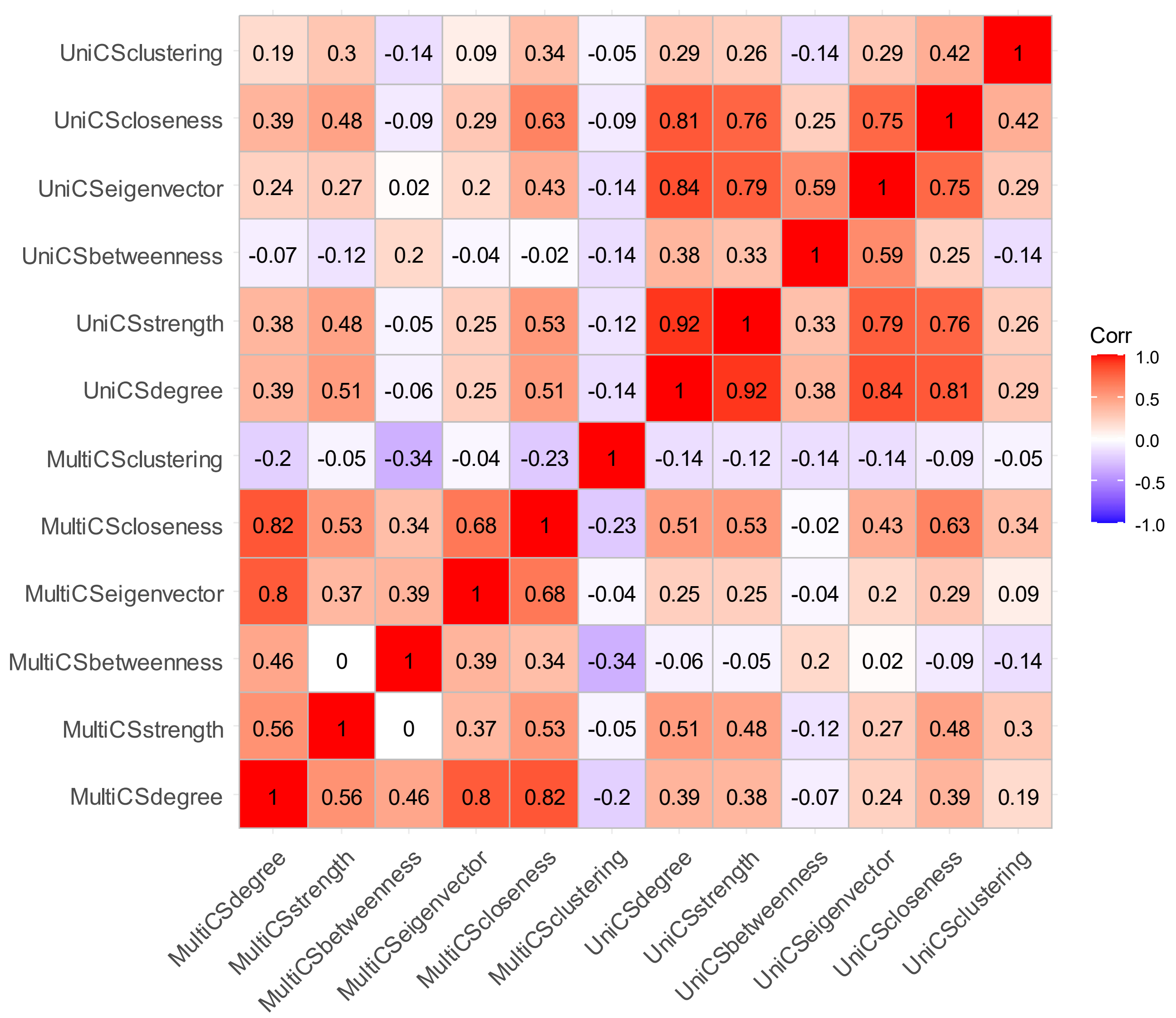
Corr
1.0
0.5
0.0
-0.5
-1.0
UniCSclustering
UniCScloseness
UniCSeigenvector
UniCSbetweenness
UniCSstrength
UniCSdegree
MultiCSclustering
MultiCScloseness
MultiCSeigenvector
MultiCSbetweenness
MultiCSstrength
MultiCSdegree
0.19 0.3 -0.14 0.09 0.34 -0.05 0.29 0.26 -0.14 0.29 0.42 1
0.39 0.48 -0.09 0.29 0.63 -0.09 0.81 0.76 0.25 0.75 1 0.42
0.24 0.27 0.02 0.2 0.43 -0.14 0.84 0.79 0.59 1 0.75 0.29
-0.07 -0.12 0.2 -0.04 -0.02 -0.14 0.38 0.33 1 0.59 0.25 -0.14
0.38 0.48 -0.05 0.25 0.53 -0.12 0.92 1 0.33 0.79 0.76 0.26
0.39 0.51 -0.06 0.25 0.51 -0.14 1 0.92 0.38 0.84 0.81 0.29
-0.2 -0.05 -0.34 -0.04 -0.23 1 -0.14 -0.12 -0.14 -0.14 -0.09 -0.05
0.82 0.53 0.34 0.68 1 -0.23 0.51 0.53 -0.02 0.43 0.63 0.34
0.8 0.37 0.39 1 0.68 -0.04 0.25 0.25 -0.04 0.2 0.29 0.09
0.46 0 1 0.39 0.34 -0.34 -0.06 -0.05 0.2 0.02 -0.09 -0.14
0.56 1 0 0.37 0.53 -0.05 0.51 0.48 -0.12 0.27 0.48 0.3
1 0.56 0.46 0.8 0.82 -0.2 0.39 0.38 -0.07 0.24 0.39 0.19
MultiCSdegree
MultiCSstrength
MultiCSbetweenness
MultiCSeigenvector
MultiCScloseness
MultiCSclustering
UniCSdegree
UniCSstrength
UniCSbetweenness
UniCSeigenvector
UniCScloseness
UniCSclustering

Figure S4: Histograms of edge weights. Multi: Multiplex affiliation network. Uni: Uniplex affiliation network.

Group A

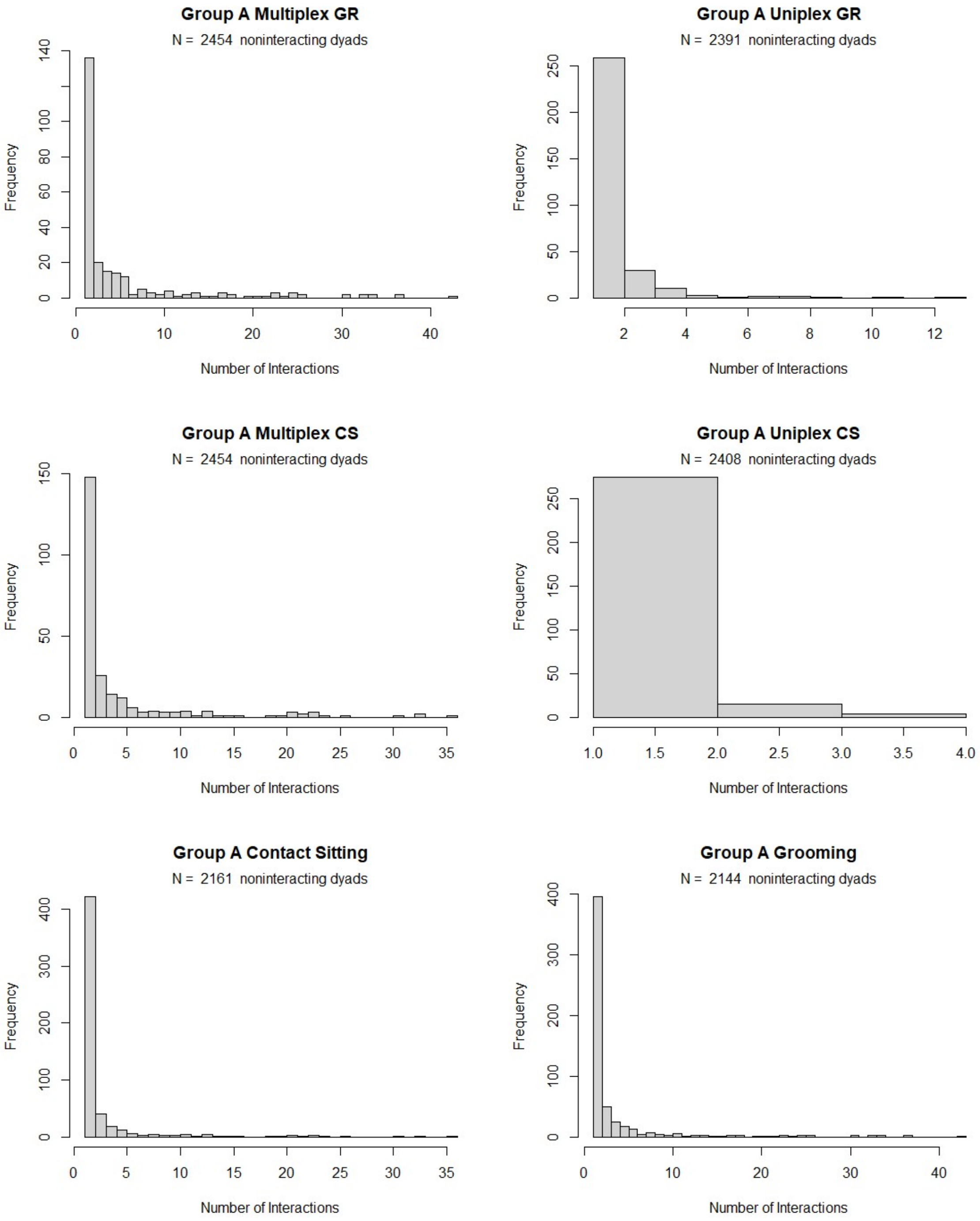

Group B

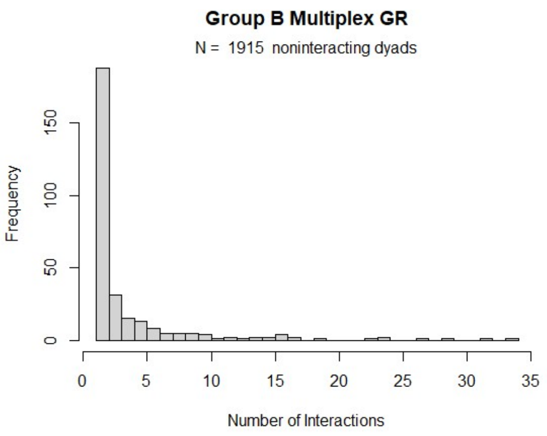
Group B Multiplex GR
N = 1915 noninteracting dyads
Frequency
Number of Interactions

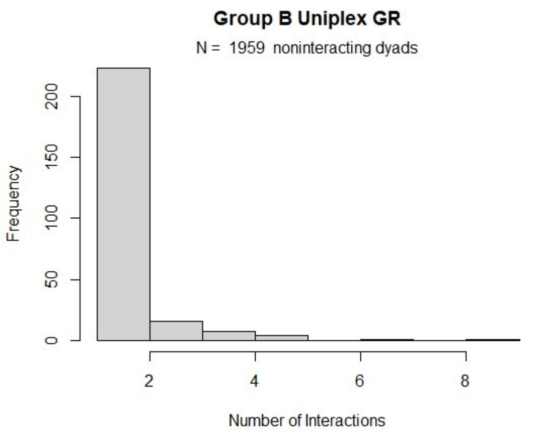
Group B Uniplex GR
N = 1959 noninteracting dyads
Frequency
Number of Interactions

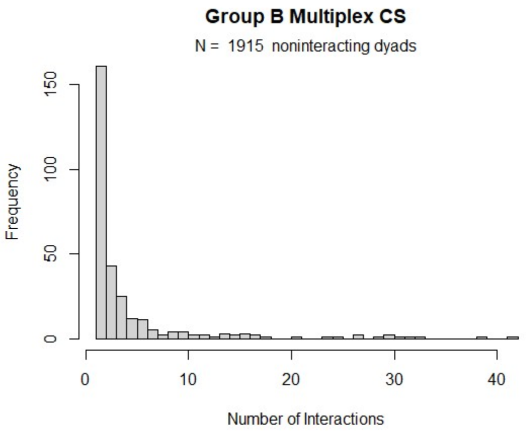
Group B Multiplex CS
N = 1915 noninteracting dyads
Frequency
Number of Interactions

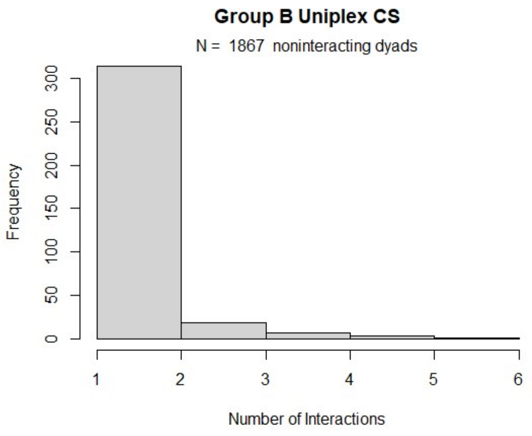
Group B Uniplex CS
N = 1867 noninteracting dyads
Frequency
Number of Interactions

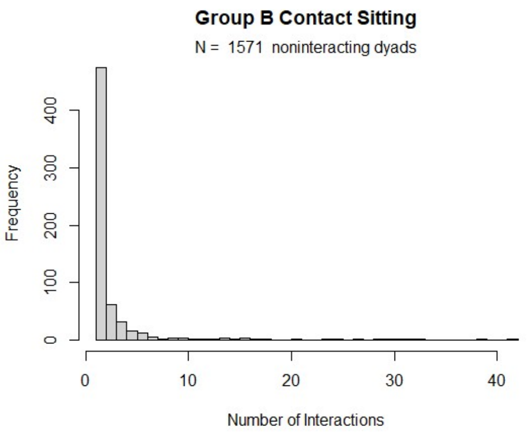
Group B Contact Sitting
N = 1571 noninteracting dyads
Frequency
Number of Interactions

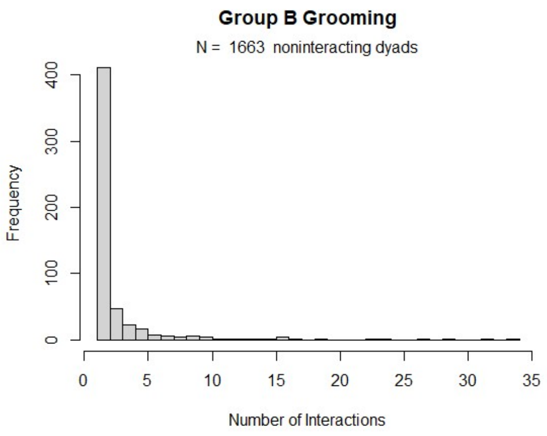
Group B Grooming
N = 1663 noninteracting dyads
Frequency
Number of Interactions

Group C

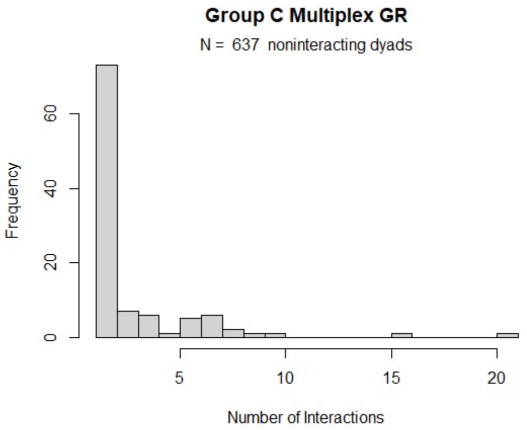
Group C Multiplex GR
N = 637 noninteracting dyads
Frequency
Number of Interactions

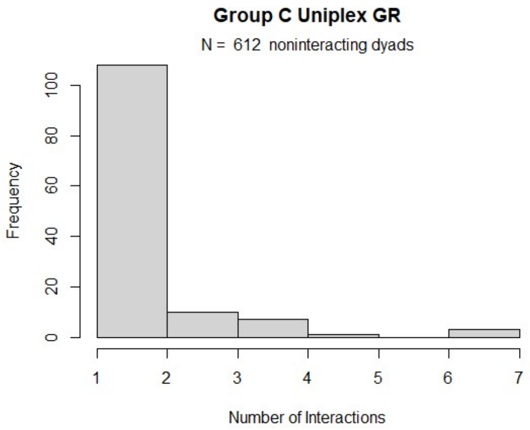
Group C Uniplex GR
N = 612 noninteracting dyads
Frequency
Number of Interactions

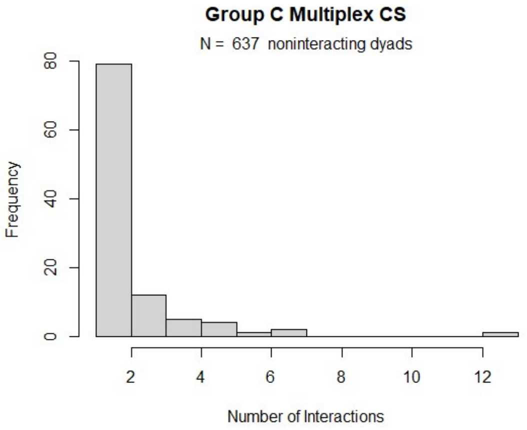
Group C Multiplex CS
N = 637 noninteracting dyads
Frequency
Number of Interactions

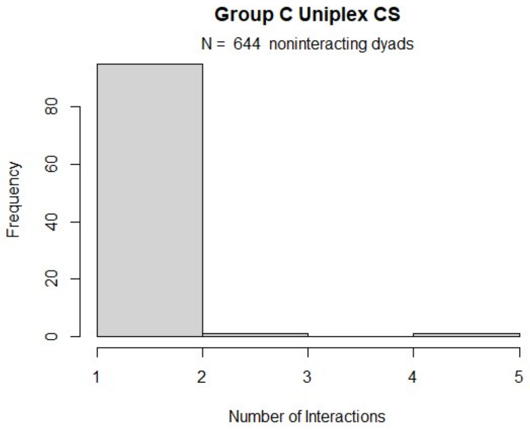
Group C Uniplex CS
N = 644 noninteracting dyads
Frequency
Number of Interactions

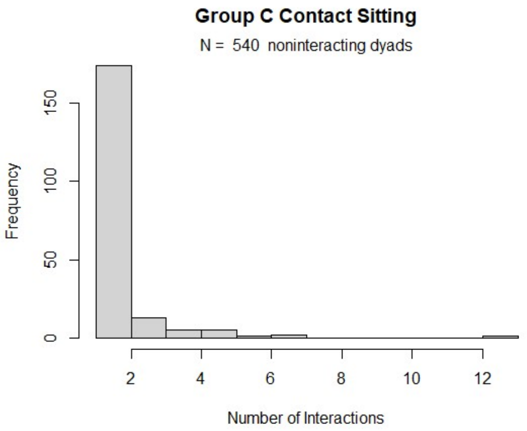
Group C Contact Sitting
N = 540 noninteracting dyads
Frequency
Number of Interactions

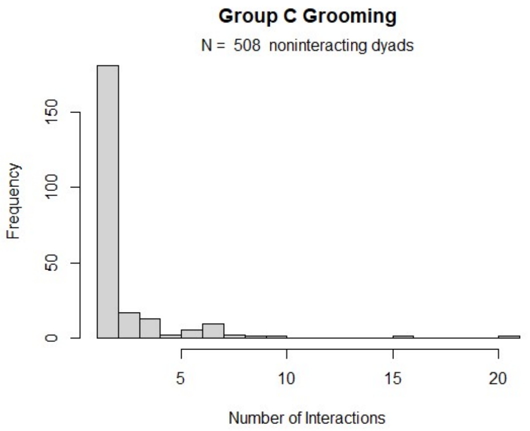
Group C Grooming
N = 508 noninteracting dyads
Frequency
Number of Interactions

Group D

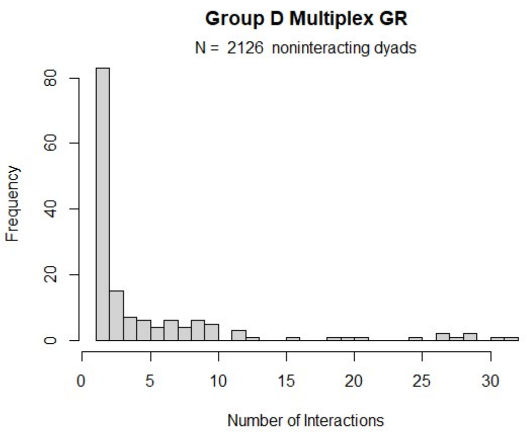
Group D Multiplex GR
N = 2126 noninteracting dyads
Frequency
Number of Interactions

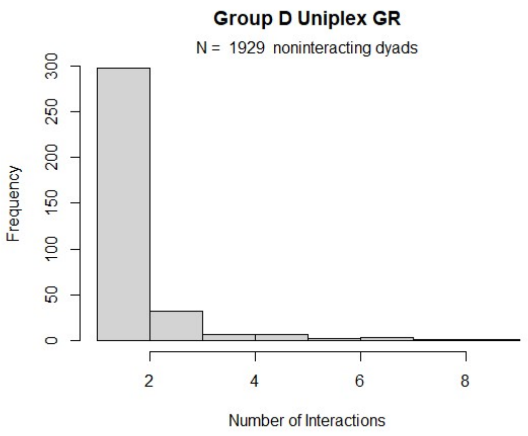
Group D Uniplex GR
N = 1929 noninteracting dyads
Frequency
Number of Interactions

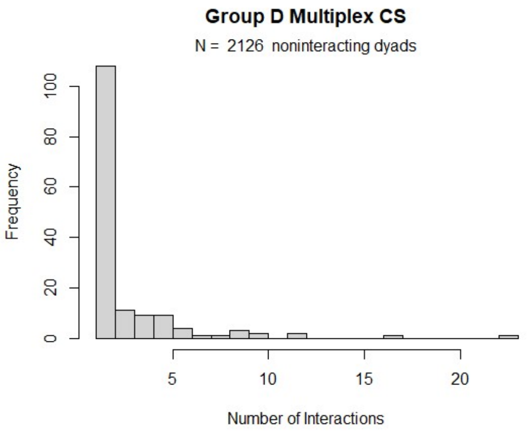
Group D Multiplex CS
N = 2126 noninteracting dyads
Frequency
Number of Interactions

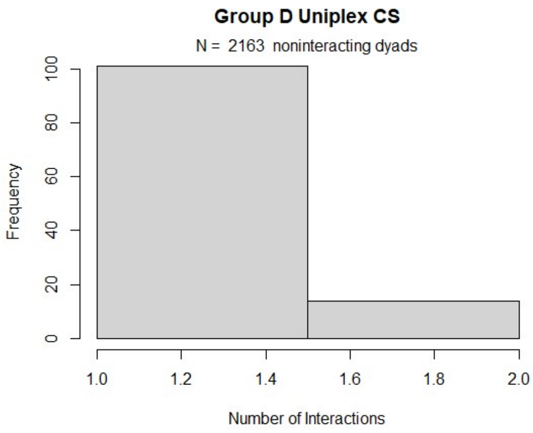
Group D Uniplex CS
N = 2163 noninteracting dyads
Frequency
Number of Interactions

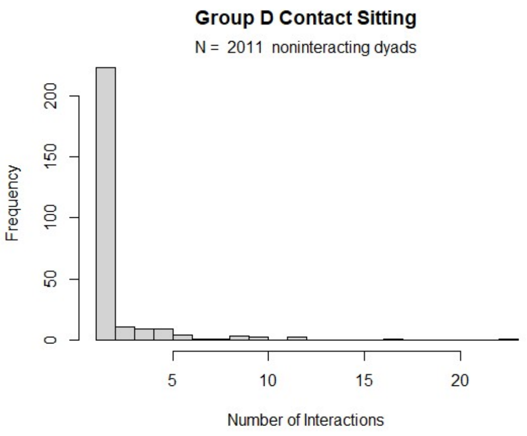
Group D Contact Sitting
N = 2011 noninteracting dyads
Frequency
Number of Interactions

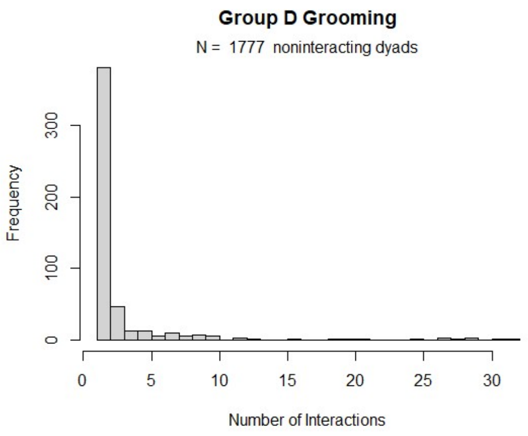
Group D Grooming
N = 1777 noninteracting dyads
Frequency
Number of Interactions

Table S1: Network Node and Edge Details by Group

Group	Multi GR		Uni GR		Multi CS		Uni CS		All Grooming		Contact Sit	
	N (female)	# interaction	N (female)	# interaction	N (female)	# interaction	N (female)	# interaction	N (female)	# interaction	N (female)	# interaction
Group A	74	1373	73	521	74	1085	74	372	74	1894	74	1457
Group B	67	1094	67	392	67	1319	67	485	67	1486	67	1804
Group C	39	288	39	211	39	206	39	114	39	499	39	320
Group D	68	776	68	575	68	404	62	129	68	1351	68	533

Table S2: Paired comparisons of whole network metrics

	Multi v Uni Groom		Multi v Uni Contact Sit		Groom v. Contact sit	
	t	p	t	p	t	p
Density	0.55	0.62	0.35	0.75	-2.20	0.12
Modularity	-5.33	0.01	-5.15	0.01	11.82	0.001
Eigenvector Centralization	-2.81	0.07	-0.55	0.62	0.41	0.71
Avg Edge Weight	-4.64	0.02	-3.66	0.04	-4.69	0.02
Clustering Coefficient	-8.75	0.003	-4.13	0.03	1.43	0.25
Reciprocity	-6.83	0.006	-	-	-	-
Proportion Kin	-5.78	0.01	-4.44	0.02	1.08	0.36
Proportion Up Rank	5.04	0.02	-	-	-	-
Rank Disparity	8.90	0.003	9.26	0.003	0.12	0.91
Rank/Eigenvector centrality correlation	0.68	0.55	-1.92	0.15	1.38	0.26

t-statistics are from paired t-tests with df = 3.

Table S3: Model Building Log for IL-6

	Model	Random	N	# Params	AIC	dAIC
**1. Establishing Relevant Covariates**						
1	Empty Model	Cage	247	1	1535.2	0
2	age	Cage	247	2	1535.4	0.1
6	samplingorder	Cage	247	2	1536.34	1.1
3	dominancecertainty	Cage	247	2	1537.2	2.0
4	percentiledominancerank	Cage	247	2	1537.2	2.0
5	percentiledominancerank*dominancecertainty	Cage	247	4	1541.1	8.9
**2. Network Model Fitting**						
71	MultiGRcloseness + UniGRcloseness	Cage	247	3	1525.09	0.00
67	MultiGRdegree + UniGRcloseness	Cage	247	3	1527.68	2.59
72	MultiGRclustering + UniGRcloseness	Cage	247	3	1528.13	3.04
70	MultiGReigenvector + UniGRcloseness	Cage	247	3	1528.64	3.55
69	MultiGRbetweenness + UniGRcloseness	Cage	247	3	1528.68	3.59
68	MultiGRstrength + UniGRcloseness	Cage	247	3	1528.69	3.61
65	MultiGRcloseness + UniGReigenvector	Cage	247	3	1530.29	5.20
47	MultiGRcloseness + UniGRdegree	Cage	247	3	1530.49	5.40
53	MultiGRcloseness + UniGRstrength	Cage	247	3	1530.63	5.55
66	MultiGRclustering + UniGReigenvector	Cage	247	3	1530.96	5.87
48	MultiGRclustering + UniGRdegree	Cage	247	3	1531.45	6.36
40	AllGReigenvector + AllCSclustering	Cage	247	3	1531.55	6.46
54	MultiGRclustering + UniGRstrength	Cage	247	3	1531.67	6.58
61	MultiGRdegree + UniGReigenvector	Cage	247	3	1531.69	6.60
43	MultiGRdegree + UniGRdegree	Cage	247	3	1531.77	6.68
16	AllGReigenvector + AllCSstrength	Cage	247	3	1531.86	6.77
63	MultiGRbetweenness + UniGReigenvector	Cage	247	3	1531.91	6.83
49	MultiGRdegree + UniGRstrength	Cage	247	3	1532.01	6.92
45	MultiGRbetweenness + UniGRdegree	Cage	247	3	1532.09	7.00
64	MultiGReigenvector + UniGReigenvector	Cage	247	3	1532.19	7.10
62	MultiGRstrength + UniGReigenvector	Cage	247	3	1532.19	7.10
51	MultiGRbetweenness + UniGRstrength	Cage	247	3	1532.24	7.15
44	MultiGRstrength + UniGRdegree	Cage	247	3	1532.38	7.29
46	MultiGReigenvector + UniGRdegree	Cage	247	3	1532.41	7.32
50	MultiGRstrength + UniGRstrength	Cage	247	3	1532.47	7.39
52	MultiGReigenvector + UniGRstrength	Cage	247	3	1532.49	7.40
13	AllGRdegree + AllCSstrength	Cage	247	3	1532.61	7.52
22	AllGReigenvector + AllCSbetweenness	Cage	247	3	1532.64	7.55
41	AllGRcloseness + AllCSclustering	Cage	247	3	1533.17	8.08
17	AllGRcloseness + AllCSstrength	Cage	247	3	1533.17	8.08
37	AllGRdegree + AllCSclustering	Cage	247	3	1533.18	8.09
34	AllGReigenvector + AllCScloseness	Cage	247	3	1533.27	8.18
19	AllGRdegree + AllCSbetweenness	Cage	247	3	1533.38	8.29
23	AllGRcloseness + AllCSbetweenness	Cage	247	3	1533.78	8.69
21	AllGRbetweenness + AllCSbetweenness	Cage	247	3	1533.90	8.81
10	AllGReigenvector + AllCSdegree	Cage	247	3	1534.22	9.13
28	AllGReigenvector + AllCSeigenvector	Cage	247	3	1534.26	9.17

39	AllGRbetweenness + AllCSclustering	Cage	247	3	1534.29	9.20
35	AllGRcloseness + AllCScloseness	Cage	247	3	1534.49	9.40
15	AllGRbetweenness + AllCSstrength	Cage	247	3	1534.57	9.48
14	AllGRstrength + AllCSstrength	Cage	247	3	1534.59	9.50
59	MultiGRcloseness + UniGRbetweenness	Cage	247	3	1534.65	9.56
24	AllGRclustering + AllCSbetweenness	Cage	247	3	1534.75	9.67
60	MultiGRclustering + UniGRbetweenness	Cage	247	3	1534.76	9.67
92	MultiCSstrength + UniCSbetweenness	Cage	247	3	1534.79	9.70
20	AllGRstrength + AllCSbetweenness	Cage	247	3	1534.81	9.72
31	AllGRdegree + AllCScloseness	Cage	247	3	1534.95	9.86
57	MultiGRbetweenness + UniGRbetweenness	Cage	247	3	1535.36	10.27
25	AllGRdegree + AllCSeigenvector	Cage	247	3	1535.37	10.28
11	AllGRcloseness + AllCSdegree	Cage	247	3	1535.37	10.28
29	AllGRcloseness + AllCSeigenvector	Cage	247	3	1535.38	10.29
7	AllGRdegree + AllCSdegree	Cage	247	3	1535.43	10.34
80	MultiCSstrength + UniCSdegree	Cage	247	3	1535.56	10.48
38	AllGRstrength + AllCSclustering	Cage	247	3	1535.75	10.66
58	MultiGReigenvector + UniGRbetweenness	Cage	247	3	1535.80	10.72
55	MultiGRdegree + UniGRbetweenness	Cage	247	3	1535.83	10.74
56	MultiGRstrength + UniGRbetweenness	Cage	247	3	1535.88	10.79
18	AllGRclustering + AllCSstrength	Cage	247	3	1535.94	10.85
27	AllGRbetweenness + AllCSeigenvector	Cage	247	3	1536.01	10.93
98	MultiCSstrength + UniCSeigenvector	Cage	247	3	1536.05	10.96
33	AllGRbetweenness + AllCScloseness	Cage	247	3	1536.20	11.11
110	MultiCSstrength + UniCSclustering	Cage	247	3	1536.20	11.11
9	AllGRbetweenness + AllCSdegree	Cage	247	3	1536.28	11.19
42	AllGRclustering + AllCSclustering	Cage	247	3	1536.28	11.20
114	MultiCSclustering + UniCSclustering	Cage	247	3	1536.52	11.44
93	MultiCSbetweenness + UniCSbetweenness	Cage	247	3	1536.56	11.47
86	MultiCSstrength + UniCSstrength	Cage	247	3	1536.62	11.53
111	MultiCSbetweenness + UniCSclustering	Cage	247	3	1536.68	11.59
96	MultiCSclustering + UniCSbetweenness	Cage	247	3	1536.70	11.61
104	MultiCSstrength + UniCScloseness	Cage	247	3	1536.91	11.82
30	AllGRclustering + AllCSeigenvector	Cage	247	3	1536.96	11.87
94	MultiCSeigenvector + UniCSbetweenness	Cage	247	3	1536.97	11.88
108	MultiCSclustering + UniCScloseness	Cage	247	3	1537.02	11.93
78	MultiGRclustering + UniGRclustering	Cage	247	3	1537.12	12.03
105	MultiCSbetweenness + UniCScloseness	Cage	247	3	1537.15	12.06
26	AllGRstrength + AllCSeigenvector	Cage	247	3	1537.19	12.10
112	MultiCSeigenvector + UniCSclustering	Cage	247	3	1537.28	12.19
84	MultiCSclustering + UniCSdegree	Cage	247	3	1537.34	12.25
102	MultiCSclustering + UniCSeigenvector	Cage	247	3	1537.36	12.27
90	MultiCSclustering + UniCSstrength	Cage	247	3	1537.43	12.34
75	MultiGRbetweenness + UniGRclustering	Cage	247	3	1537.44	12.35
81	MultiCSbetweenness + UniCSdegree	Cage	247	3	1537.48	12.39
99	MultiCSbetweenness + UniCSeigenvector	Cage	247	3	1537.51	12.42
87	MultiCSbetweenness + UniCSstrength	Cage	247	3	1537.58	12.49
12	AllGRclustering + AllCSdegree	Cage	247	3	1537.61	12.52
95	MultiCScloseness + UniCSbetweenness	Cage	247	3	1537.64	12.55
36	AllGRclustering + AllCScloseness	Cage	247	3	1537.75	12.66
8	AllGRstrength + AllCSdegree	Cage	247	3	1537.79	12.71

91	MultiCSdegree + UniCSbetweenness	Cage	247	3	1537.80	12.71
32	AllGRstrength + AllCScloseness	Cage	247	3	1537.87	12.78
109	MultiCSdegree + UniCSclustering	Cage	247	3	1537.93	12.84
113	MultiCScloseness + UniCSclustering	Cage	247	3	1538.02	12.93
106	MultiCSeigenvector + UniCScloseness	Cage	247	3	1538.05	12.96
82	MultiCSeigenvector + UniCSdegree	Cage	247	3	1538.16	13.07
76	MultiGReigenvector + UniGRclustering	Cage	247	3	1538.21	13.12
100	MultiCSeigenvector + UniCSeigenvector	Cage	247	3	1538.21	13.12
88	MultiCSeigenvector + UniCSstrength	Cage	247	3	1538.38	13.29
83	MultiCScloseness + UniCSdegree	Cage	247	3	1538.73	13.64
101	MultiCScloseness + UniCSeigenvector	Cage	247	3	1538.77	13.68
79	MultiCSdegree + UniCSdegree	Cage	247	3	1538.84	13.75
97	MultiCSdegree + UniCSeigenvector	Cage	247	3	1538.87	13.78
103	MultiCSdegree + UniCScloseness	Cage	247	3	1538.91	13.82
73	MultiGRdegree + UniGRclustering	Cage	247	3	1538.94	13.86
77	MultiGRcloseness + UniGRclustering	Cage	247	3	1538.95	13.86
107	MultiCScloseness + UniCScloseness	Cage	247	3	1538.99	13.90
74	MultiGRstrength + UniGRclustering	Cage	247	3	1539.02	13.93
85	MultiCSdegree + UniCSstrength	Cage	247	3	1539.14	14.05
89	MultiCScloseness + UniCSstrength	Cage	247	3	1539.14	14.05

Table S4: Model Results from top models for IL-6

	Model Number																	
**Parameters**	71	67	72	70	69	68	65	47	53	66	48	40	54	61	43	16	63	49
Intercept	-0.102	-1.06	-0.68	-0.64	-0.91	-0.94	2.72 **	2.61 **	2.76 **	1.79 **	1.76 **	2.13 **	1.85 **	1.72 **	1.70 **	1.87 **	1.59 **	1.81 **
Uni GR Closeness	7.92 **	7.39 **	6.31 **	6.80 **	6.53 **	6.63 **	-	-	-	-	-	-	-	-	-	-	-	-
Uni GR Eigenvec.	-	-	-	-	-	-	1.58 **	-	-	1.24 *	-	-	-	1.44 **	-	-	1.21 *	-
Uni GR Degree	-	-	-	-	-	-	-	0.08 **	-	-	0.07 *	-	-	-	0.08 **	-	-	-
Uni GR Strength	-	-	-	-	-	-	-	-	15.18 **	-	-	-	11.92 *	-	-	-	-	14.12 **
Multi GR Closeness	-3.68 *	-	-	-	-	-	-3.16	-2.93	-2.97	-	-	-	-	-	-	-	-	-
Multi GR Degree	-	-0.04	-	-	-	-	-	-	-	-	-	-	-	-0.03	-0.03	-	-	-0.03
Multi GR Clustering	-	-	-0.36	-	-	-	-	-	-	-0.53	-0.47	-	-0.44	-	-	-	-	-
Multi GR Eigenvec.	-	-	-	-0.11	-	-	-	-	-	-	-	-	-	-	-	-	-	-
Multi GR Between.	-	-	-	-	0.46	-	-	-	-	-	-	-	-	-	-	-	2.01	-
Multi GR Strength	-	-	-	-	-	-0.02	-	-	-	-	-	-	-	-	-	-	-	-
All GR Eigenvec.	-	-	-	-	-	-	-	-	-	-	-	1.09 *	-	-	-	1.14 *	-	-
All CS Clustering	-	-	-	-	-	-	-	-	-	-	-	-1.75 +	-	-	-	-	-	-
All CS Strength	-	-	-	-	-	-	-	-	-	-	-	-	-	-	-	-3.72	-	-
ΔAIC	0	2.59	3.04	3.55	3.59	3.61	5.2	5.4	5.55	5.87	6.36	6.46	6.58	6.6	6.68	6.77	6.83	6.92

+ p < 0.1; * p < 0.05; ** p < 0.01

Table S5: Model Building Log for TNF-α

	Model	Random	N	# Params	AIC	dAIC
**1. Establishing Relevant Covariates**						
1	Empty Model	Cage	248	1	2653.25	0.00
3	dominancecertainty	Cage	248	2	2654.61	1.36
5	percentiledominancerank*dominancecertainty	Cage	248	4	2654.70	1.45
6	samplingorder	Cage	248	2	2654.80	1.55
4	percentiledominancerank	Cage	248	2	2654.89	1.64
2	age	Cage	248	2	2655.03	1.78
**2. Network Model Fitting**						
53	MultiGRcloseness + UniGRstrength	Cage	248	3	2641.17	0.00
49	MultiGRdegree + UniGRstrength	Cage	248	3	2642.76	1.59
54	MultiGRclustering + UniGRstrength	Cage	248	3	2644.94	3.77
34	AllGReigenvector + AllCScloseness	Cage	248	3	2645.47	4.31
59	MultiGRcloseness + UniGRbetweenness	Cage	248	3	2645.85	4.68
47	MultiGRcloseness + UniGRdegree	Cage	248	3	2646.06	4.89
43	MultiGRdegree + UniGRdegree	Cage	248	3	2646.82	5.65
35	AllGRcloseness + AllCScloseness	Cage	248	3	2646.91	5.74
10	AllGReigenvector + AllCSdegree	Cage	248	3	2647.05	5.89
52	MultiGReigenvector + UniGRstrength	Cage	248	3	2647.07	5.90
65	MultiGRcloseness + UniGReigenvector	Cage	248	3	2647.46	6.29
33	AllGRbetweenness + AllCScloseness	Cage	248	3	2647.72	6.55
48	MultiGRclustering + UniGRdegree	Cage	248	3	2648.03	6.87
71	MultiGRcloseness + UniGRcloseness	Cage	248	3	2648.28	7.11
7	AllGRdegree + AllCSdegree	Cage	248	3	2648.35	7.18
14	AllGRstrength + AllCSstrength	Cage	248	3	2648.45	7.29
32	AllGRstrength + AllCScloseness	Cage	248	3	2648.46	7.30
8	AllGRstrength + AllCSdegree	Cage	248	3	2648.49	7.32
55	MultiGRdegree + UniGRbetweenness	Cage	248	3	2648.50	7.34
11	AllGRcloseness + AllCSdegree	Cage	248	3	2648.68	7.51
31	AllGRdegree + AllCScloseness	Cage	248	3	2648.71	7.54
61	MultiGRdegree + UniGReigenvector	Cage	248	3	2648.82	7.65
9	AllGRbetweenness + AllCSdegree	Cage	248	3	2648.86	7.69
51	MultiGRbetweenness + UniGRstrength	Cage	248	3	2649.07	7.90
50	MultiGRstrength + UniGRstrength	Cage	248	3	2649.23	8.06
60	MultiGRclustering + UniGRbetweenness	Cage	248	3	2649.75	8.58
66	MultiGRclustering + UniGReigenvector	Cage	248	3	2649.84	8.67
72	MultiGRclustering + UniGRcloseness	Cage	248	3	2650.10	8.93
46	MultiGReigenvector + UniGRdegree	Cage	248	3	2650.21	9.05
67	MultiGRdegree + UniGRcloseness	Cage	248	3	2650.45	9.28
28	AllGReigenvector + AllCSeigenvector	Cage	248	3	2650.83	9.66
36	AllGRclustering + AllCScloseness	Cage	248	3	2651.23	10.06
12	AllGRclustering + AllCSdegree	Cage	248	3	2651.27	10.10
16	AllGReigenvector + AllCSstrength	Cage	248	3	2651.32	10.16
58	MultiGReigenvector + UniGRbetweenness	Cage	248	3	2651.33	10.16
64	MultiGReigenvector + UniGReigenvector	Cage	248	3	2651.40	10.24
44	MultiGRstrength + UniGRdegree	Cage	248	3	2651.46	10.29

15	AllGRbetweenness + AllCSstrength	Cage	248	3	2651.66	10.49
45	MultiGRbetweenness + UniGRdegree	Cage	248	3	2651.81	10.64
13	AllGRdegree + AllCSstrength	Cage	248	3	2652.10	10.93
89	MultiCScloseness + UniCSstrength	Cage	248	3	2652.13	10.96
62	MultiGRstrength + UniGReigenvector	Cage	248	3	2652.33	11.16
27	AllGRbetweenness + AllCSeigenvector	Cage	248	3	2652.38	11.22
56	MultiGRstrength + UniGRbetweenness	Cage	248	3	2652.40	11.23
17	AllGRcloseness + AllCSstrength	Cage	248	3	2652.48	11.31
18	AllGRclustering + AllCSstrength	Cage	248	3	2652.49	11.32
107	MultiCScloseness + UniCScloseness	Cage	248	3	2652.54	11.37
26	AllGRstrength + AllCSeigenvector	Cage	248	3	2652.55	11.38
29	AllGRcloseness + AllCSeigenvector	Cage	248	3	2652.59	11.42
70	MultiGReigenvector + UniGRcloseness	Cage	248	3	2652.66	11.49
57	MultiGRbetweenness + UniGRbetweenness	Cage	248	3	2652.73	11.57
83	MultiCScloseness + UniCSdegree	Cage	248	3	2652.73	11.57
63	MultiGRbetweenness + UniGReigenvector	Cage	248	3	2652.75	11.58
90	MultiCSclustering + UniCSstrength	Cage	248	3	2652.76	11.59
85	MultiCSdegree + UniCSstrength	Cage	248	3	2652.84	11.67
87	MultiCSbetweenness + UniCSstrength	Cage	248	3	2652.88	11.71
68	MultiGRstrength + UniGRcloseness	Cage	248	3	2652.89	11.72
108	MultiCSclustering + UniCScloseness	Cage	248	3	2652.91	11.74
103	MultiCSdegree + UniCScloseness	Cage	248	3	2652.97	11.80
86	MultiCSstrength + UniCSstrength	Cage	248	3	2653.05	11.88
105	MultiCSbetweenness + UniCScloseness	Cage	248	3	2653.10	11.93
69	MultiGRbetweenness + UniGRcloseness	Cage	248	3	2653.16	11.99
25	AllGRdegree + AllCSeigenvector	Cage	248	3	2653.20	12.03
104	MultiCSstrength + UniCScloseness	Cage	248	3	2653.21	12.04
88	MultiCSeigenvector + UniCSstrength	Cage	248	3	2653.31	12.15
84	MultiCSclustering + UniCSdegree	Cage	248	3	2653.32	12.16
81	MultiCSbetweenness + UniCSdegree	Cage	248	3	2653.38	12.21
79	MultiCSdegree + UniCSdegree	Cage	248	3	2653.40	12.23
101	MultiCScloseness + UniCSeigenvector	Cage	248	3	2653.46	12.29
106	MultiCSeigenvector + UniCScloseness	Cage	248	3	2653.58	12.41
80	MultiCSstrength + UniCSdegree	Cage	248	3	2653.64	12.47
82	MultiCSeigenvector + UniCSdegree	Cage	248	3	2653.84	12.67
102	MultiCSclustering + UniCSeigenvector	Cage	248	3	2654.08	12.91
97	MultiCSdegree + UniCSeigenvector	Cage	248	3	2654.09	12.93
38	AllGRstrength + AllCSclustering	Cage	248	3	2654.17	13.00
98	MultiCSstrength + UniCSeigenvector	Cage	248	3	2654.22	13.05
99	MultiCSbetweenness + UniCSeigenvector	Cage	248	3	2654.32	13.15
39	AllGRbetweenness + AllCSclustering	Cage	248	3	2654.35	13.18
20	AllGRstrength + AllCSbetweenness	Cage	248	3	2654.36	13.19
40	AllGReigenvector + AllCSclustering	Cage	248	3	2654.58	13.41
30	AllGRclustering + AllCSeigenvector	Cage	248	3	2654.68	13.52
21	AllGRbetweenness + AllCSbetweenness	Cage	248	3	2654.69	13.52
100	MultiCSeigenvector + UniCSeigenvector	Cage	248	3	2654.73	13.56
22	AllGReigenvector + AllCSbetweenness	Cage	248	3	2654.73	13.56
42	AllGRclustering + AllCSclustering	Cage	248	3	2655.24	14.07
77	MultiGRcloseness + UniGRclustering	Cage	248	3	2655.28	14.11
113	MultiCScloseness + UniCSclustering	Cage	248	3	2655.33	14.16
95	MultiCScloseness + UniCSbetweenness	Cage	248	3	2655.33	14.17

37	AllGRdegree + AllCSclustering	Cage	248	3	2655.39	14.22
41	AllGRcloseness + AllCSclustering	Cage	248	3	2655.45	14.28
24	AllGRclustering + AllCSbetweenness	Cage	248	3	2655.57	14.40
23	AllGRcloseness + AllCSbetweenness	Cage	248	3	2655.65	14.48
19	AllGRdegree + AllCSbetweenness	Cage	248	3	2655.71	14.54
78	MultiGRclustering + UniGRclustering	Cage	248	3	2655.79	14.62
96	MultiCSclustering + UniCSbetweenness	Cage	248	3	2655.90	14.73
114	MultiCSclustering + UniCSclustering	Cage	248	3	2655.91	14.74
92	MultiCSstrength + UniCSbetweenness	Cage	248	3	2656.05	14.88
110	MultiCSstrength + UniCSclustering	Cage	248	3	2656.07	14.91
91	MultiCSdegree + UniCSbetweenness	Cage	248	3	2656.30	15.13
73	MultiGRdegree + UniGRclustering	Cage	248	3	2656.37	15.20
109	MultiCSdegree + UniCSclustering	Cage	248	3	2656.46	15.29
93	MultiCSbetweenness + UniCSbetweenness	Cage	248	3	2656.86	15.69
74	MultiGRstrength + UniGRclustering	Cage	248	3	2656.93	15.76
111	MultiCSbetweenness + UniCSclustering	Cage	248	3	2656.96	15.79
75	MultiGRbetweenness + UniGRclustering	Cage	248	3	2656.99	15.82
94	MultiCSeigenvector + UniCSbetweenness	Cage	248	3	2657.05	15.88
76	MultiGReigenvector + UniGRclustering	Cage	248	3	2657.14	15.97
112	MultiCSeigenvector + UniCSclustering	Cage	248	3	2657.16	15.99

Table S6: Model Results from top models for TNF-α

	Model Number												
**Parameters**	53	49	54	34	59	47	43	35	10	52	65	33	48
Intercept	6.53 **	4.83 **	3.90 **	8.31 **	7.13 **	6.30 **	4.82 **	5.12 **	5.08 **	4.52 **	6.42 **	3.25 **	4.01 **
Uni GR Strength	23.81 **	25.36 **	20.61 **	-	-	-	-	-	-	21.09	-	-	-
Uni GR Between.	-	-	-	-	22.90 **	-	-	-	-	-	-	-	-
Uni GR Degree	-	-	-	-	-	0.09 **	0.10 **	-	-	-	-	-	0.08 **
Uni GR Eigenvec.	-	-	-	-	-	-	-	-	-	-	1.66 **	-	-
Uni GR Closeness	-	-	-	-	-	-	-	-	-	-	-	3.81 *	-
Multi GR Closeness	-6.26 **	-	-	-	-6.64 **	-5.29 **	-	-	-	-	-5.52 *	-	-
Multi GR Degree	-	-0.12 **	-	-	-	-	-0.10 *	-	-	-	-	-	-
Multi GR Clustering	-	-	1.04 *	-	-	-	-	-	-	-	-	-	0.94 +
Multi GR Eigenvec.	-	-	-	-	-	-	-	-	-	-0.85	-	-	-
Multi GR Between.	-	-	-	-	-	-	-	-	-	-	-	0.15	-
All GR Eigenvec.	-	-	-	1.68 **	-	-	-	-	1.50 *	-	-	-	-
All GR Closeness	-	-	-	-	-	-	-	7.45 *	-	-	-	-	-
All CS Closeness	-	-	-	-8.00 **	-	-	-	-8.08 **	-	-	-	-	-
All CS Degree	-	-	-	-	-	-	-	-	-0.07 **	-	-	-	-
ΔAIC	0	1.59	3.77	4.31	4.68	4.89	5.65	5.74	5.89	5.9	6.29	6.55	6.87

+ $p < 0.1$; * $p < 0.05$; ** $p < 0.01$
